# hsa_circ_0063865 regulates cytoskeleton rearrangement and endoplasmic reticulum stress to promote esophageal tumorigenesis under co-exposure to N-nitrosamines and microcystin

**DOI:** 10.1186/s12967-026-08554-8

**Published:** 2026-07-07

**Authors:** Mingjun Sun, Zhikui Gao, Ping Ye, Yuan Ding, Jia Wang, Tingyu Li, Xianghu Wang, Yadong Feng, Lihua Ren, Yong Zhu, Ruihua Shi, Ran Liu

**Affiliations:** 1 Engineering Research Center of Small Molecule Drugs, Ministry of Education, School of Public Health, Guangdong Pharmaceutical University, Guangzhou, Guangdong Province 510006 China; 2 Key Laboratory of Environmental Medicine Engineering, School of Public Health, Ministry of Education, Southeast University, Nanjing, China; 3 Yunnan Provincial Key Laboratory of Public Health and Biosafety, Kunming, China; 4 Department of Gastroenterology, School of Medicine, Zhongda Hospital, Southeast University, Nanjing, China; 5 School of Medicine, Taizhou University, Taizhou, China; 6 Winthrop P. Rockefeller Cancer Institute, University of Arkansas for Medical Sciences, Little Rock, AR USA

**Keywords:** Esophageal squamous cell carcinoma, hsa_circ_0063865, N-Nitrosamines and microcystins, Cytoskeleton remodeling, MYH9^+^ epithelial cell

## Abstract

**Background:**

High enrichment of N-nitrosamines (NAs) and microcystins (MCs) in drinking water are major risk factors in high-risk areas of esophageal squamous cell carcinoma (ESCC) in China, however, their combined effects and underlying carcinogenic mechanisms remain unknown.

**Methods:**

The scRNA_seq sequencing and CTD database were used to identify the “chemical-epithelial cell” associated carcinogenic targets. An ESCC rat model was established to confirm the combined effect of NAs and MCs; RNA-Seq was performed to screen potential circRNAs in ESCC and matched adjacent normal tissues. Malignant transformation of esophageal epithelial cells (Het-1A-T) was induced by exposure to N-nitrosomethylbenzylamine (NMBzA) and Microcystin-LR (MC-LR). The biological function of hsa_circ_0063865 was investigated using gain- and loss-of-function experiments in vitro and in vivo. RNA pull-down, ChIRP, RIP, Co-IP and luciferase reporter assays were used to elucidate the underlying mechanisms of hsa_circ_0063865 in chemically related ESCC.

**Results:**

The rat ESCC model and median-effect principle showed a synergistic effect of NAs and MC-LR in chemical carcinogenesis. Further, integration of single-cell and network toxicology revealed 292 “chemical-epithelial cell” associated carcinogenic targets, which were primarily involved in microRNA in cancer and cytoskeleton rearrangements. At the molecular level, the chemical carcinogenesis-related hsa_circ_0063865 was identified and found to be up-regulated in ESCC tissues. Functionally, hsa_circ_0063865 promoted malignant transformation of Het-1A cells induced by NMBzA and MC-LR by promoting cytoskeletal rearrangements and inhibiting ER stress of MYH9^+^ epithelial cell. Mechanistically, hsa_circ_0063865 acted as a modular scaffold to tether eEF1A2(126-177nt of hsa_circ_0063865) and NMIIA (601-658nt of hsa_circ_0063865), thereby promoting NMIIA translation by enhancing the interaction between eEF1A2 and NMIIA, which further promoted cytoskeletal reorganization; Additionally, hsa_circ_0063865 competitively upregulated RCN1 expression by sponging miR-450b-3p, resulting in ER stress inhibition and depolarization of mitochondrial membrane potential via activation of the PERK-eIF2α-ATF4-CHOP axis in MYH9^+^ epithelial cell.

**Conclusion:**

These findings identify a novel chemical carcinogenic target and elucidate the dual-regulatory mechanism of hsa_circ_0063865 in NAs and MC-LR-induced esophageal carcinogenesis.

**Supplementary Information:**

The online version contains supplementary material available at https://doi.org/10.1186/s12967-026-08554-8.

## Graphical abstract

**Figure:**
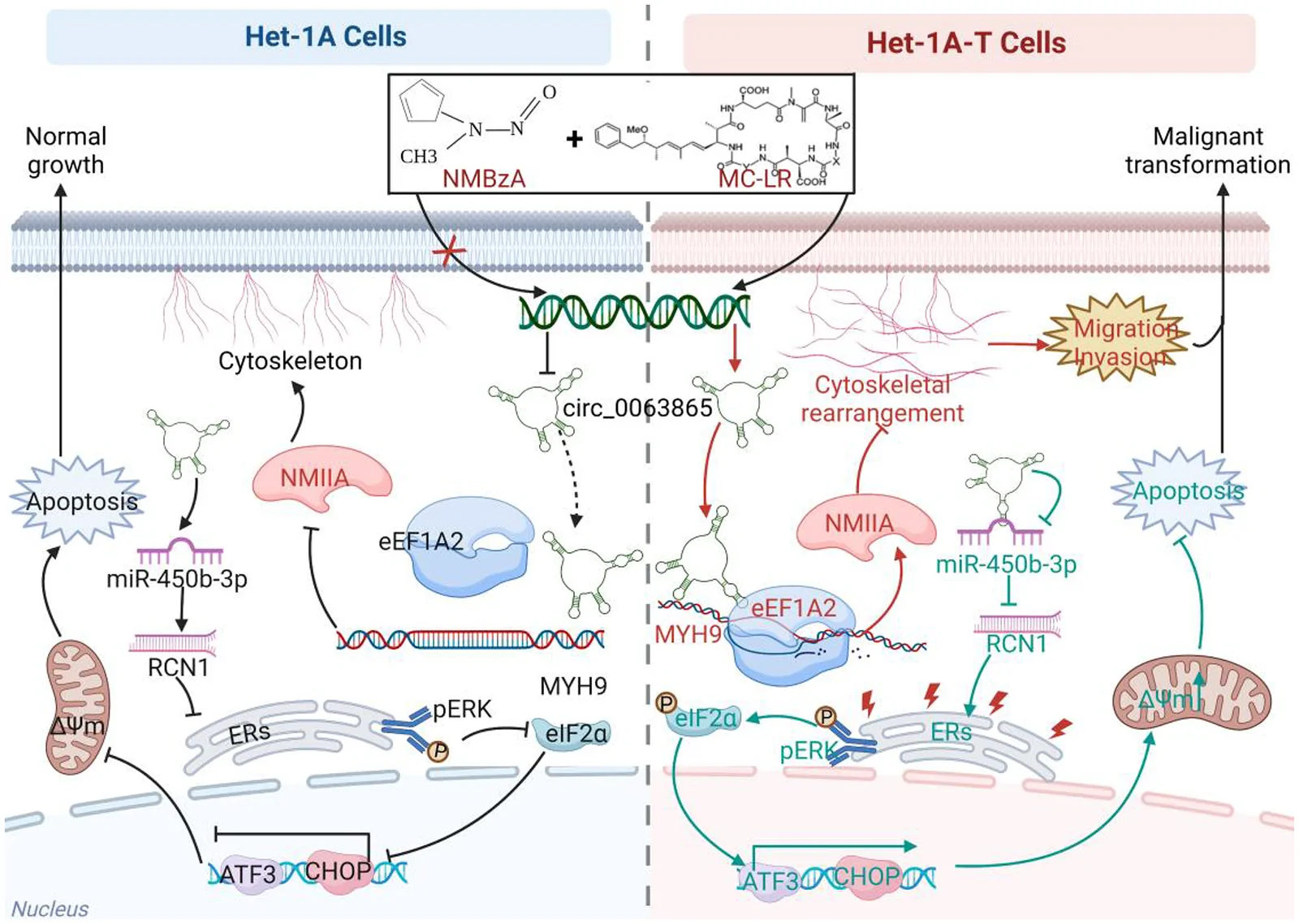

## Introduction

Esophageal cancer (EC), one of the most prevalent upper gastrointestinal cancers, imposes a substantial global cancer burden, particularly in Eastern Asia and Southern Africa. Annually, over 604,000 new EC cases are diagnosed worldwide, of which esophageal squamous cell carcinoma (ESCC) accounts for approximately 90%, and the overall five-year survival rate of ESCC patients remains below 20% [1]. Therefore, early screening and elimination of ESCC risk factors are necessary, however, the current understanding of ESCC etiology remains incomplete. Notably, our previous epidemiological survey in Huai’an, a high-incidence area of ESCC in China, suggested that water pollution may be an important contributor to ESCC risk, accounting for 54.3% of the observed risk [2]. Therefore, it is urgent to conduct further research on environmental risk factors responsible for the high morbidity and mortality in Huai’an to reduce ESCC incidence.

In recent decades, human activities, especially industrial and agricultural production, have inevitably resulted in the deposition of ammonia-nitrogen compounds and eutrophication in source water. Unfortunately, the widespread use of chlorinating disinfectants in drinking water treatment can induce the formation of nitrosamine disinfection by-products (DBPs), for instance, the maximum mass concentration of N-nitrosodimethylamine was 160ng/L in distribution pipe networks in Canada, whereas the concentration in raw water was below the detection limit (0.4-1.6ng/L) [3]. In Huai’an, higher concentrations of N-nitrosodipropylamine (NDPA; 34.91 ± 32.7ng/L) and N-nitrosodiethylamine(NDEA; 36.53 ± 65.31ng/L) were detected in the drinking water [4]. Even worse, the concentration of Microcystin-LR (MC-LR) in raw water in Huai’an was also much higher than that in low-risk regions of ESCC [5]. MC-LR is a cyclic hepatotoxic peptides that has been implicated in the development of liver, colorectal and gastric cancer through various pathways, including induction of estrogenic effects [6], destruction of cytoskeleton [7] and metabolic disorders [8]. So far, however, the relationship between MC-LR and the risk of ESCC has not been conclusive. Importantly, our previous studies in esophageal epithelial cells and rat models suggested that NAs may induce malignant transformation of esophageal epithelial cells, while MC-LR may further promote this process [9]. However, the potential molecular mechanisms underlying the combined effects of NAs and MC-LR in ESCC development are still poorly understood.

As a class of endogenous RNA, circular RNAs (circRNAs) are widely expressed in tissues of humans. In recent years, considerable studies have revealed that the circular transcripts can even be expressed at levels dozens of times higher than the linear transcripts, indicating circRNAs are not meaninglessly by-products produced by RNA splicing [10]. Further, it is also gradually clarified that the biogenesis, localization, degradation and modification of specific circRNAs, and the interaction relationship with DNA, RNA, and proteins, remain incompletely understood, but the localization patterns and function of most annotated circRNAs remain unknown. During the course of various disease processes such as tumorigenesis, many circRNAs served as natural miRNA sponges, and through competitively binding to miRNAs, the inhibition of mRNAs induced by the same was reduced, e.g. circ_0000326/miR-338-3p/RAB14 [11], circHMGCS1/miR-503-5p/IGF2 [12] and circ_001783/miR-200c-3p/ZEB1 [13], etc. In addition, emerging evidence has revealed that aberrantly expressed circRNAs can serve as protein decoys, scaffolds and recruiters to mediate cancer progression by regulating the translation and splicing of downstream proteins. For example, Liu et al. reported that circRNA-MTO1 interacted with TRAF4, and sequestered TRAF4 from activating Eg5 translation, thereby reducing the Eg5 protein level [14]. Wang et al. found that circRHOT1 can initiate NR2F6 expression via recruiting TIP60 to promote hepatocellular carcinoma progression [15]. Notably, the potential roles of circRNAs in chemical carcinogenesis have attracted considerable attention recently. For example, hsa_circ_0006916/miR-522-3p/TNRC6A has been shown to initiate BPDE-induced malignant transformation of 16HBE cells [16]. In addition, hsa_circ_0009048, which is regulated by QKI hypermethylation, attenuates lung tumorigenesis induced by NNK [17]. Moreover, circCIMT silencing promotes cadmium-induced malignant transformation of lung epithelial cells through the DNA base excision repair pathway [18]. To date, the role of circRNAs in NAs and MC-LR-induced ESCC carcinogenesis, and the mechanisms involved, remain unclear.

In the present study, we performed the RNA-seq to screen the differentially expressed circRNAs in ESCC and adjacent non-tumor tissues and identified the upregulated hsa_circ_0063865, which was also confirmed using the malignant transformed Het-1A cells induced by N-nitrosomethylbenzylamine (NMBzA) and microcystin-LR (MC-LR). Then, we further identified the functions and targets of hsa_circ_0063865 in the carcinogenic process of NMBzA and MC-LR by the loss- and gain-of-function experiments. We demonstrated that hsa_circ_0063865 promoted malignant transformation of Het-1A cells induced by exposure to NMBzA and MC-LR through promoting cell proliferation, invasion, migration, and inhibiting early apoptosis in vitro and in vivo. Mechanistically, hsa_circ_0063865 could as a molecular bridge and promote translation of NMIIA through facilitating the interaction between eEF1A2 and NMIIA, which further promoted cytoskeletal reorganization. These results contribute to an understanding of the processes involved in carcinogenesis caused by NAs and MC-LR.

## Results

### NAs combined with MC-LR induced esophageal carcinogenesis by regulating cytoskeletal rearrangement and endoplasmic reticulum stress in epithelial cell subsets

**Fig. 1:**
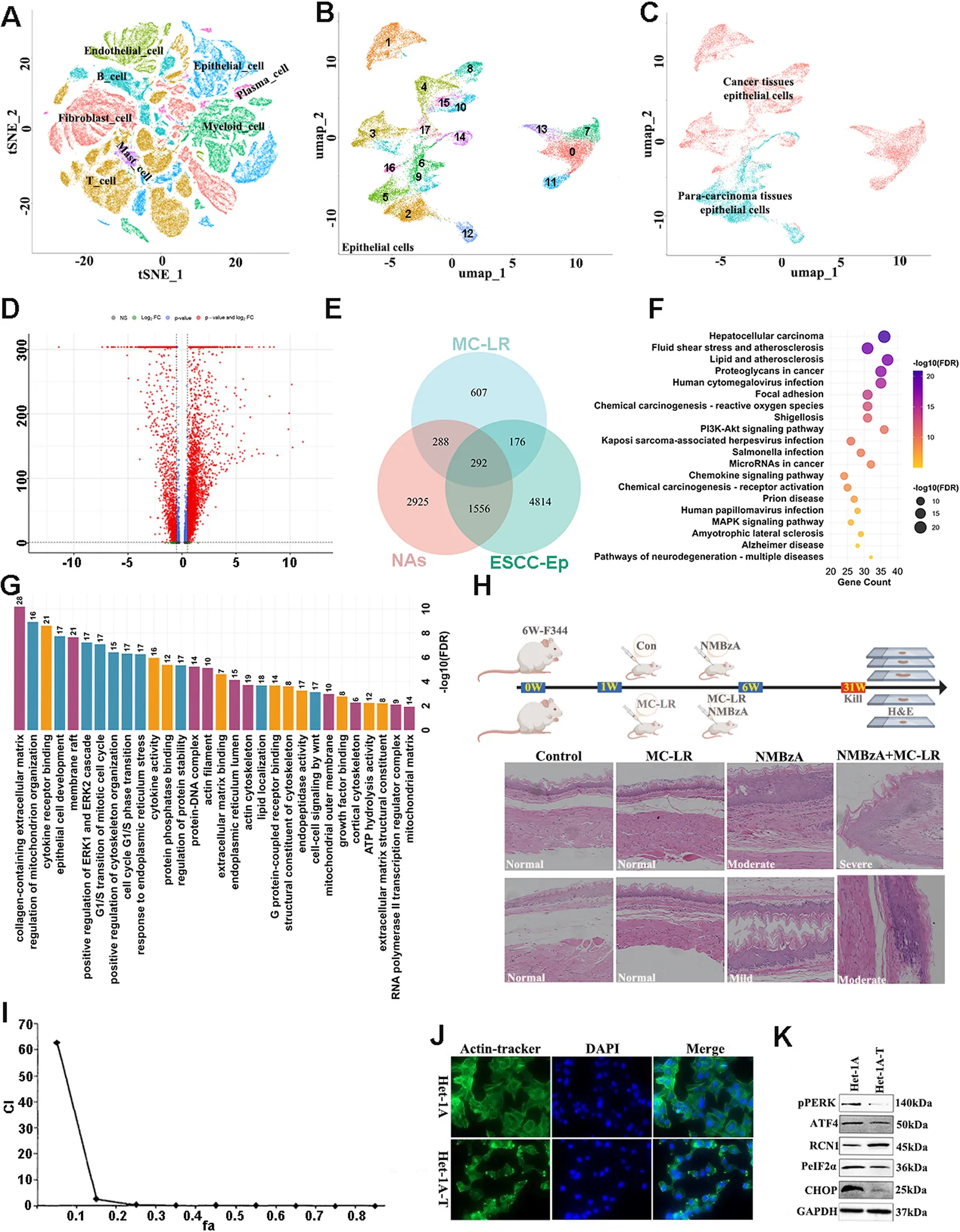
NAs combined with MC-LR induced esophageal carcinogenesis by regulating cytoskeletal rearrangement and endoplasmic reticulum stress of epithelial cell subsets. **(A)** All 8 cell subtypes in ESCC were annotated according to marker genes. **(B)** The UMAP algorithm divided the 23,459 epithelial cells into 18 clusters. **(C)** The UMAP algorithm divided 18 clusters of epithelial cells into Cancer tissues-epithelial cells and Para-carcinoma tissues-epithelial cells. **(D)** The volcano map of differentially expressed genes. **(E)** A total of 292 overlapping genes were identified as “chemical-epithelial cell” related carcinogenic genes. **(F)** KEGG enrichment and **(G)**GO enrichment analysis of core targets. **(H)** Schematic drawing of the rat model of ESCC. **(I)** The line graphs of moderate efficiency principle. **(J)** Comparative analysis of cell cytoskeleton between Het-1A and Het-1A-T. **(K)**The protein levels of ER stress pathway in Het-1A and Het-1A-T

Our study analyzed 123,929 cells derived from 10 ESCC patient recruited in Huaian, Jiangsu. After quality control, data filtering, and normalization, we identified epithelial cells, endothelial cells, mast cells, B cells, monocyte cells, T cells, fibroblasts and plasma cells based on marker genes (Fig. 1A; Fig.S1A-B). Subsequently, a total of 23,459 epithelial cells were extracted and identified as non-cancerous epithelial cells and cancerous epithelial cells (Fig. 1B&C). A volcano plot identified 6838 differentially expressed genes between the two groups (Fig. 1D). To explore the effects of NAs and MC-LR co-exposure on epithelial cells, we identified 1363 MC-LR targets and 4861 nitrosamines targets from CTD and ChEMBL databases. By integrating these gene sets, a total of 292“chemical-epithelial cell” related carcinogenic targets were identified (Fig. 1E). KEGG and GO enrichment analyses suggested that these targets were mainly involved in Chemical carcinogenesis, microRNA in cancer, positive regulation of cytoskeleton organization, endoplasmic reticulum lumen, and mitochondrial outer membrane (Fig. 1F-G). Collectively, these bioinformatics results provide hypothesis-generating evidence that NAs and MC-LR co-exposure may be associated with ER stress and cytoskeleton rearrangement.

Next, to explore the tumorigenic effect of NAs and MC-LR co-exposure, we constructed an ESCC rat model and malignant transformed cell model. As shown by the pathological results (Fig. 1H), the proportion of atypical hyperplasia was significantly higher in the NMBzA-MC-LR group than in the other groups (Table S1). Also at the cellular level, the 5 × 7 factorial design indicated that an interaction between NAs and MC-LR in ESCC-related cell growth inhibition (Table S2). Combined exposure to NMBzA and MC-LR induced a dose-dependent pattern in which cell growth inhibition initially decreased and then increased, with significant differences compared with both the control and single-exposure groups (*P* < 0.05), suggesting a possible synergistic effect (Table S3). Furthermore, median-effect analysis showed that the EC50 values of NMBzA, MC-LR, and their combined exposure were 323.619 µmol/L, 135.0 µmol/L, and 5.26 µmol/L, respectively (Table S4);Moreover, when fa = 0.3675, the CI was 0.0108, with both NMBzA and MC-LR at 100 nmol/L, demonstrating a strong synergistic effect (Fig. 1I; Table S5-7). Functionally, 100nM/L NMBzA-MC-LR arrested cells in the S phase and enhanced cell migration and invasion at 25 generations compared with the single-treated groups (Table S8-9). Subsequently, we established a malignant transformed Het-1A model (Het-1A-T) by exposing cells to NMBzA and MC-LR for 45 generations, and validated by STR sequence analysis and soft agar colony formation assay. The results showed Het-1A-T cells presented mutation at the D16S539 and FGA sites (Fig. S2A) and obtained a malignant phenotype (Fig. S2B). At the molecular level, FITC-labeled phalloidin staining revealed a disorganized cytoskeleton and enhanced pseudopod formation in Het-1A-T cells (Fig. 1J). Moreover, compared with normal Het-1A, ER stress and the related proteins p-PERK, p-EIF2α, ATF4 and CHOP were downregulated in Het-1A-T cells (Fig. 1K). These results suggest that NAs and MC-LR co-exposure can induce esophageal carcinogenesis by regulating the cytoskeleton and mitochondria and endoplasmic reticulum of epithelial cells.

### Chemical carcinogenesis-related hsa_circ_0063865 is upregulated in ESCC

**Fig. 2:**
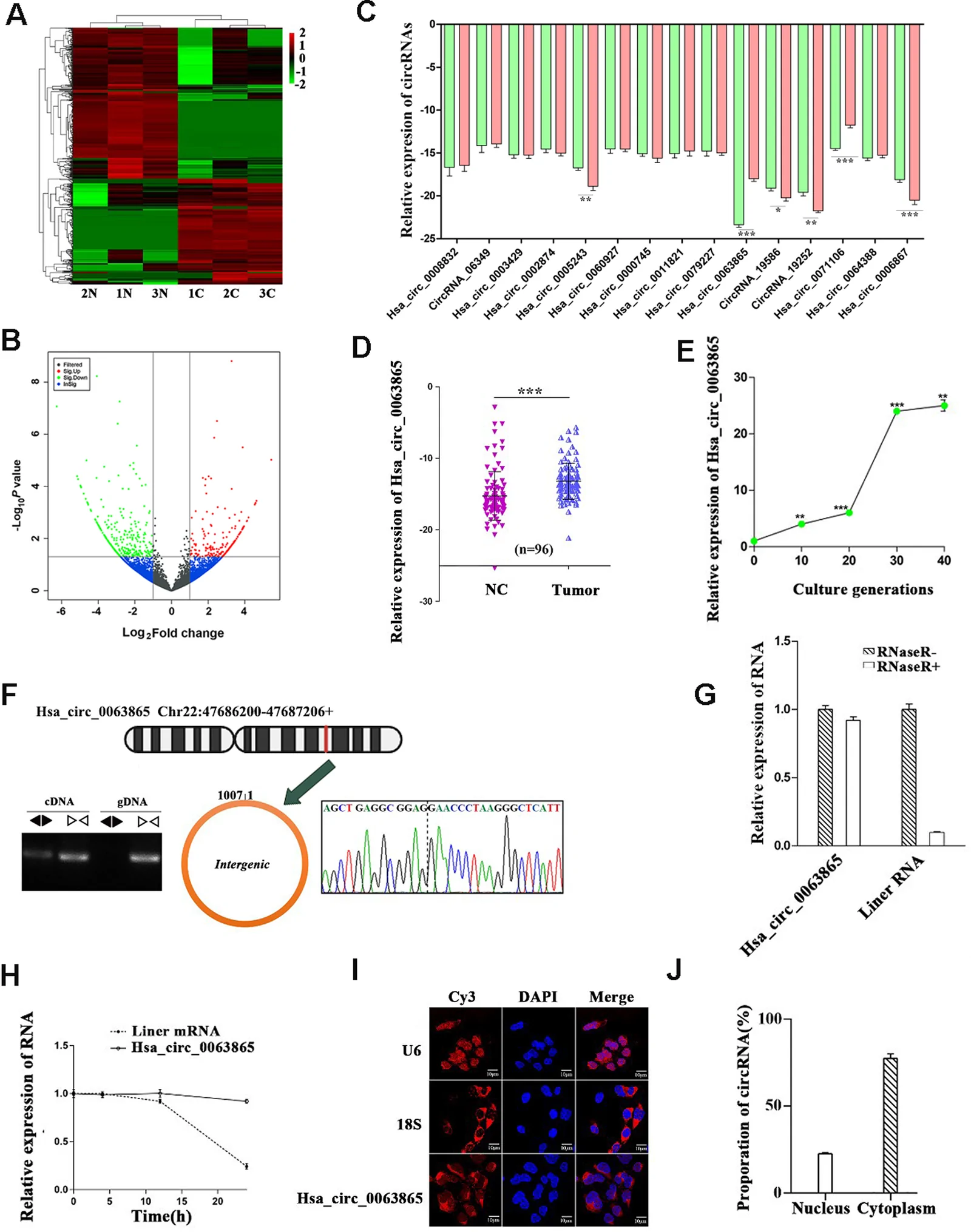
Chemical carcinogenesis-related hsa_circ_0063865 is upregulated in ESCC. **(A)** Cluster map of differentially expressed circRNAs. **(B)** The volcano map of differentially expressed circRNAs in ESCC patients. **(C)** The expression levels of 15 circRNAs in Het-1A and Het-1A-T cells. **(D)** Relative expression of hsa_circ_0063865 in ESCC tissues and adjacent normal tissues (*n* = 96). **(E)** Expressions of hsa_circ_0063865 during malignant transformation. **(F)** Schematic structure of hsa_circ_0063865 and Sanger sequencing to clarify the cyclization site. **(G)** Location analysis of hsa_circ_0063865 detected by FISH assay. **(H)** Nuclear and cytoplasmic separation assay of hsa_circ_0063865. **(I)** RNase R digestion assay of hsa_circ_0063865. **(J)** Time-dependent inhibition assay of hsa_circ_0063865 induced by Actinomycin D

Given that 292 core targets were enriched to microRNAs in cancer, we analyzed the circRNA expression profile in ESCC patients using whole transcriptomic sequencing (Fig. S3A-G). A total of 571 circRNAs with altered expression were identified in cancer tissues (Fig. 2A&B; Table S10). Further, the qRT-PCR results indicated that several circRNAs were also abnormally expressed in NAs/MC-LR-transformed cells and in 96 ESCC patients (Fig. 2C&D), and confirmed that hsa_circ_0063865 was sharply upregulated from the 20th generation to 30th generation, but tended to stabilize after 30 generations (Fig. 2E). Further analysis found that hsa_circ_0063865 overexpression in ESCC tissues was positively correlated with advanced clinical stage (I-II vs. III-IV, *P* = 0.005; Tables S11). The area under the ROC curve(AUC) was 0.809, suggesting that hsa_circ_0063865 mightserve as a biomarker for ESCC (Fig. S3H). To characterize hsa_circ_0063865, the circBase and circBank database indicated that hsa_circ_0063865 was marked as an intergenic circRNAs derived from chr22:47686200–47,687,206 with a splice length of 1007nt (Fig. 2F). Given that hsa_circ_0063865 has not been studied previously, we conducted Sanger sequencing and an RNase R exonuclease assay to verify its existence and circularization (Fig. 2G&H; Fig. S3I). Notably, FISH analysis revealed that hsa_circ_0063865 was located in both cytoplasm and nuclear (Fig. 2I), and nucleocytoplasmic separation further showed that hsa_circ_0063865 was mainly localized in cytoplasm (Fig. 2J). Taken together, these findings confirmed that the upregulation of hsa_circ_0063865 in response to long-term NAs and MC-LR co-exposure suggested its role as a tumor promoter-like circRNAs.

### hsa_circ_0063865 is involved in NAs and MC-LR co-exposure-induced malignant transformation of human esophageal epithelial cells

**Fig. 3:**
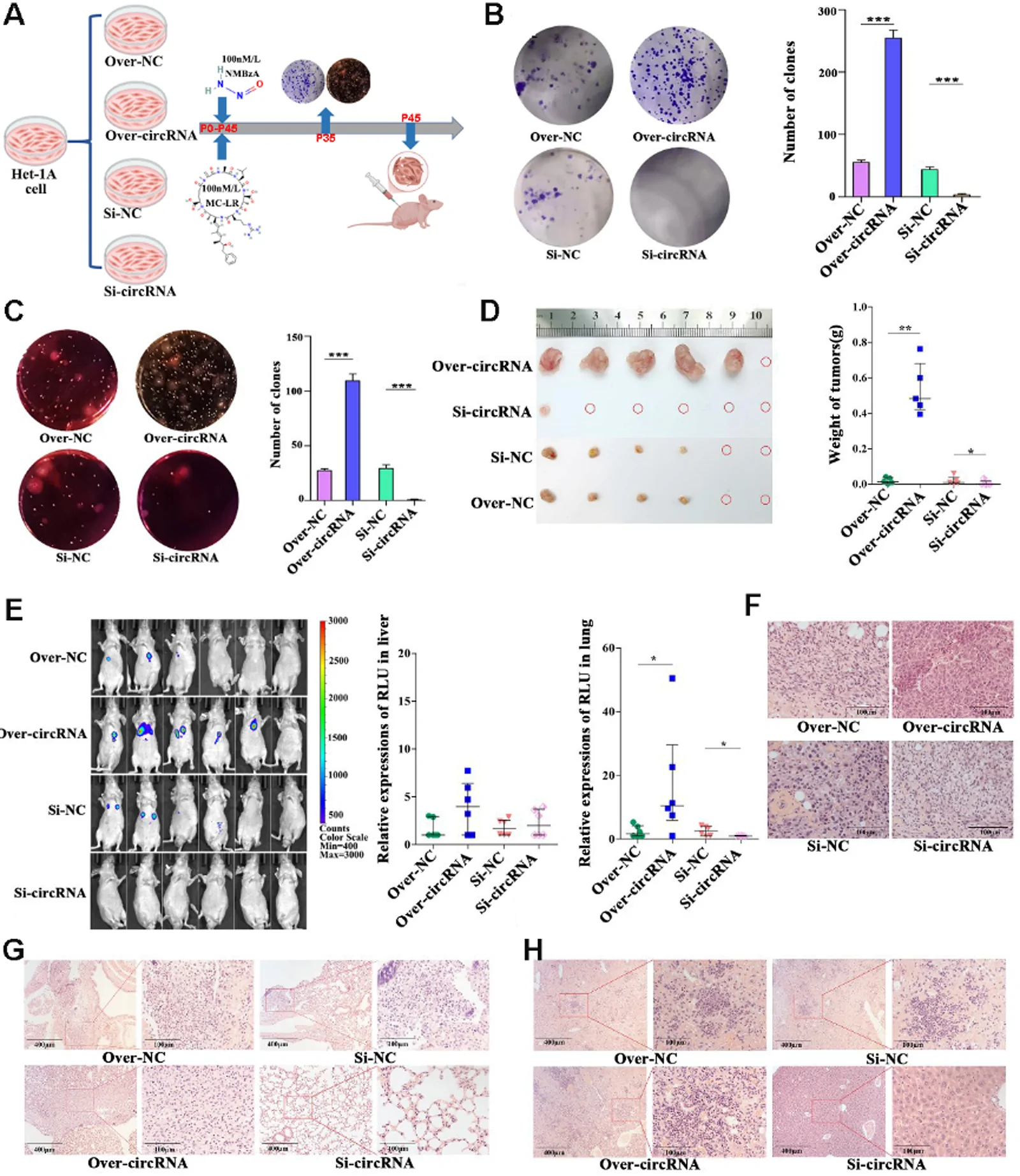
hsa_circ_0063865 is involved in NAs and MC-LR co-exposure-induced malignant transformation of human esophageal epithelial cells. **(A)** Schematic diagram of constructing malignant transformation cell model based on hsa_circ_0063865. **(B-C)** Soft agar cloning assay **(B)** and of Plate cloning assay **(C)** of 35th generation cells cultured with NMBzA and MC-LR. **(D)** Picture of subcutaneous implanted tumors. **(E)** In vivo imaging assay of metastasis tumors via tail vein injection. **(F-H)** H&E analysis of subcutaneous implanted tumors **(F)**, lung **(G)** and liver **(H)** metastasized tumors

Next, to analyze the effects of hsa_circ_0063865 on malignant transformation of Het-1A cells, we established stable cell lines over-expressing (Over-circRNA) or under-expressing (Si-circRNA) hsa_circ_0063865 (Fig.S4A&B). Subsequently, Het-1A cells transfected with Si-circRNA, Si-NC, Over-circRNA, Over-NC were cultured in medium containing NMBzA and MC-LR to trigger malignant transformation (Fig. 3A). We then detected colony-formation ability of cells cultured for 35 generations, the results showed that hsa_circ_0063865 overexpression formatted more colonies, whereas silencing hsa_circ_0063865 formatted fewer colonies in both assays compare with the controls (Fig. 3B&C). Nude mouse assays showed that hsa_circ_0063865 overexpression promoted tumorigenicity and tumor growth, whereas hsa_circ_0063865 silencing inhibited these effects (Fig. 3D;Fig. S4C-E). In the in vivo metastasis assay, IVIS imaging results suggested that Over-circRNA had more metastatic foci in both the liver or lung than the Over-NC groups, but the Si-circRNA exhibited fewer metastatic foci in the liver or lung than the Si-NC (Fig. 3E). Moreover, luciferase biochemical measurements data demonstrated that hsa_circ_0063865 over-expression promoted lung metastasis, while silencing hsa_circ_0063865 inhibited lung metastasis (Fig. 3E). Further, the H&E staining results of subcutaneous tumors (Fig. 3F), lung metastatic tumors (Fig. 3G) and liver metastatic tumors (Fig. 3H) showed that hsa_circ_0063865 increased the metastasis ability of malignant transformed cells. Together, these findings strongly confirmed that hsa_circ_0063865 promoted the tumorigenicity and metastasis of malignant transformation cells induced by MC-LR and NMBzA.

### Regulatory function of hsa_circ_0063865 in NAs combined with MC-LR-induced carcinogenesis

**Fig. 4:**
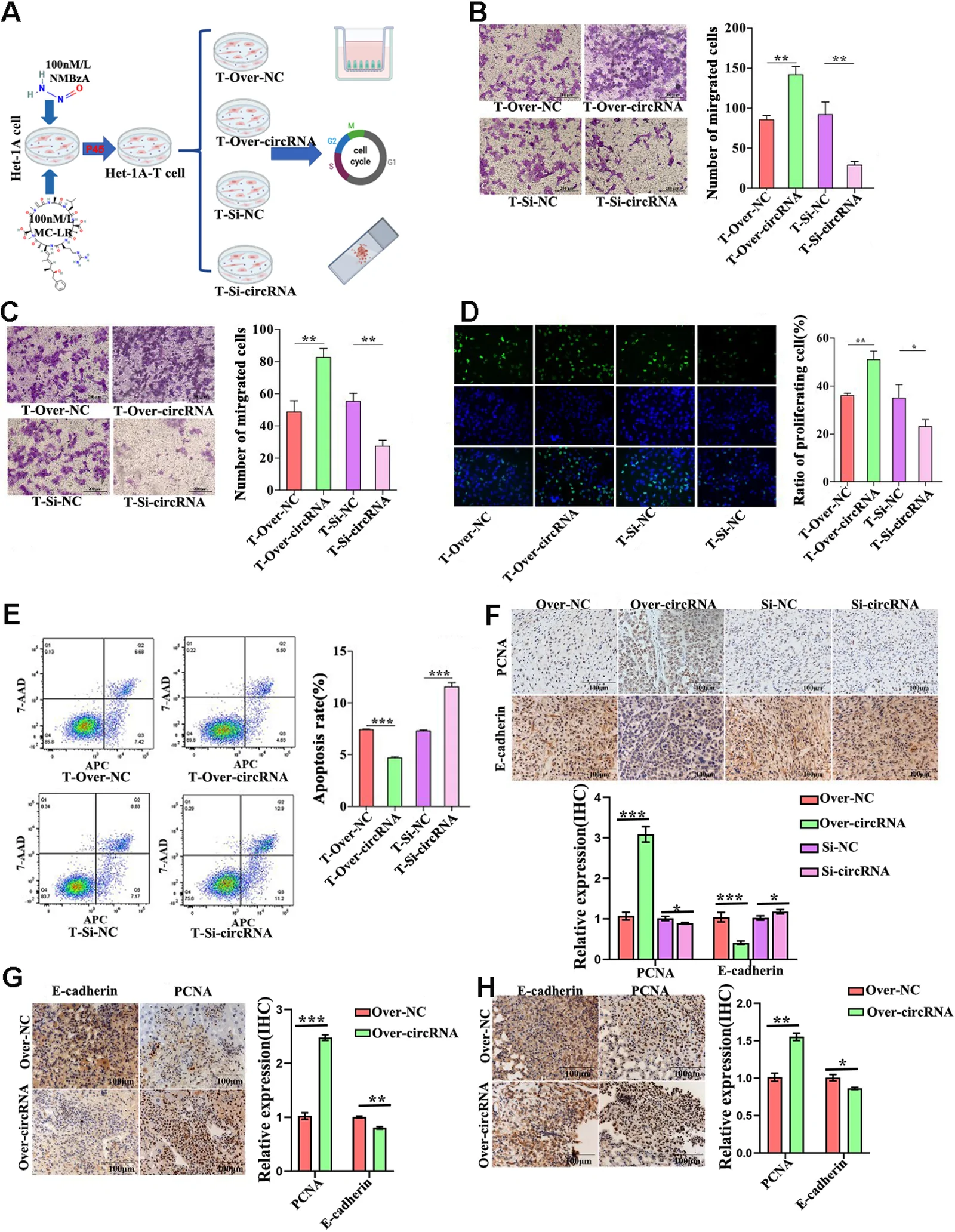
Regulatory function of hsa_circ_0063865 in NAs combined with MC-LR-induced carcinogenesis. **(A)** Schematic diagram of hsa_circ_0063865 functional exploration. **(B-C)** Effects of over-expressing/under-expressing hsa_circ_0063865 on migration **(B)** and invasion **(C)** detected by Transwell assay. **(D)** Effects of over-expressing/under-expressing hsa_circ_0063865 on proliferation of Het-1A-T were assessed EdU assay. **(E)** Effects of over-expressing/under-expressing hsa_circ_0063865 on apoptosis were detected by flow cytometry. **(F-H)** Expressions of PCNA and E-cadherin in subcutaneous implanted tumors **(F)**, liver-metastasized **(G)** and lung-metastasized **(H)** tumors were detected by IHC

Next, to investigate the functions of hsa_circ_0063865 in malignant transformed esophageal epithelial cells, we constructed Het-1A-T cells stably over-expressing and silencing hsa_circ_0063865 (Fig. 4A; Fig. S5A&B). Migration and invasion assays revealed that hsa_circ_0063865 overexpression promoted migration and invasion of Het-1A-T cells, whereas silencing hsa_circ_0063865 significantly suppressed cell migration and invasion (Fig. 4B&C). In addition, the EdU staining assay showed that hsa_circ_0063865 overexpression promoted cell proliferation, whereas the opposite result was observed in Het-1A-T cells with silencing hsa_circ_0063865 (Fig. 4D). Flow cytometric analysis demonstrated that hsa_circ_0063865 overexpression inhibited apoptosis, whereas hsa_circ_0063865 silencing increased apoptosis in Het-1A cells (Fig. 4E). To further confirm these findings in vivo, we then detected the expression of PCNA and E-cadherin in subcutaneous, lung and liver transplanted tumors by IHC staining. As shown in Fig. 4F-H, higher PCNA expression was detected in subcutaneous, lung and liver transplanted tumors overexpressing hsa_circ_0063865, whereas E-cadherin expression was upregulated in transplanted tumors with reduced hsa_circ_0063865 expression. Conversely, PCNA expression was downregulated, while E-cadherin expression was upregulated in transplanted tumors with lower hsa_circ_0063865 expression. Overall, these findings suggest that hsa_circ_0063865 act as an oncogene and promote ESCC initiation and progression.

### hsa_circ_0063865 driver cytoskeleton rearrangements and ER stress of MYH9^+^ epithelial cell by enhancing the stability of the eEF1A2-NMIIA complex

**Fig. 5:**
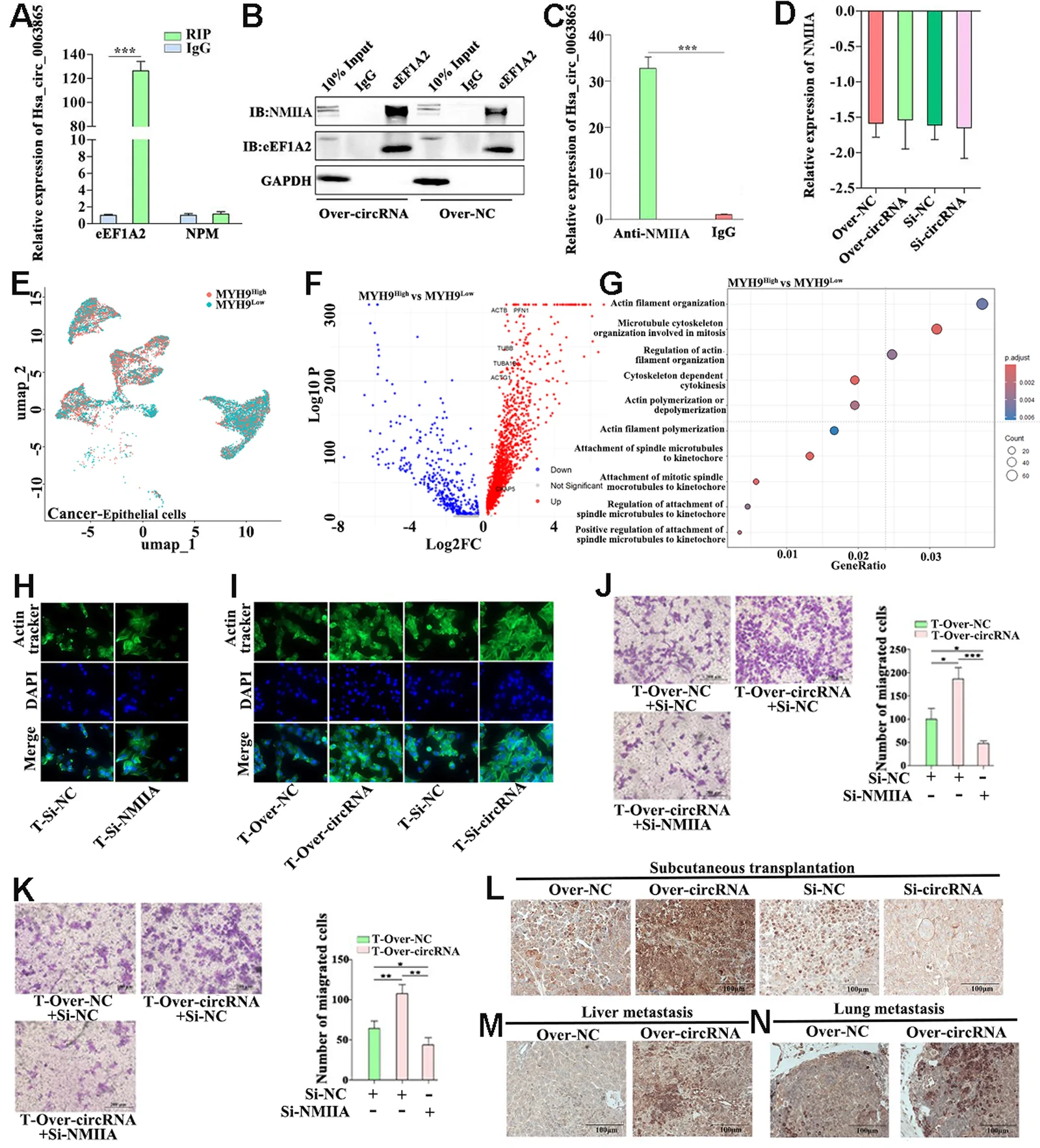
hsa_circ_0063865 driver cytoskeleton rearrangements and ER stress of MYH9^+^ epithelial cell by enhancing the stability of the eEF1A2-NMIIA complex. **(A)** Validation of combinations of identified proteins and hsa_circ_0063865 detected by RIP assay. **(B)** eEF1A2 combined more NMIIA in cells over-expressing hsa_circ_0063865 detected by Co-IP assay. **(C)** Combination of NMIIA and hsa_circ_0063865 detected by RIP assay. **(D)** Effects of over-expressing and under-expressing on mRNA expression of NMIIA detected by qRT-PCR. **(E)** The UMAP algorithm divided the Cancer tissues epithelial cells into MYH9^high^ and MYH9^Low^ cells. **(F)** The volcano map of differentially expressed genes between MYH9^high^ and MYH9^Low^ cells. **(G)** KEGG enrichment analysis of differentially expressed genes. **(H)** Inhibition of NMIIA inhibited rearrangement of cell cytoskeleton. **(I)** Phalloidin staining assay of over-expressing and under-expressing hsa_circ_0063865 on cell cytoskeleton. **(J)** hsa_circ_0063865 promoted cell migration through regulating NMIIA. **(K)** hsa_circ_0063865 promoted cell invasion through regulating NMIIA. **(L-N)** hsa_circ_0063865 promoted expression of NMIIA in subcutaneous transplanted tumors **(L)**, liver-metastasized tumors **(M)**, lung-metastasized tumor **(N)**

We further investigated the mechanism by which hsa_circ_0063865 regulated the malignant transformation of human esophageal epithelial cells, a circRNA pulldown assay was performed to identify the binding proteins of hsa_circ_0063865. Silver staining results indicated that Over-hsa_circ_0063865 pulldown group had protein bands compared to the Over-NC (Fig. S6A). The eEF1A2 and NPM proteins were detected among the hsa_circ_0063865 RNA pulldown products by mass spectrometry (Fig. S6B&C), and RIP assay verified the interaction between hsa_circ_0063865 and eEF1A2 (Fig. 5A). Subsequently, using the catRAPID database, we identified potential binding sites for the physical interaction between hsa_circ_0063865 and eEF1A2 within amino acids 50-100th of eEF1A2 (Fig. S6D). However, eEF1A is a classical G protein that delivers aminoacyl-tRNAs to the A site of the ribosome, thereby promoting the translational elongation of downstream proteins. Further, to identify eEF1A2 binding proteins, we further performed Co-IP experiments and found that eEF1A2 could bind to NMIIA (Fig. S6E&F). Remarkably, more NMIIA bound to eEF1A2 in hsa_circ_0063865 overexpression cells compared with Over-NC (Fig. 5B). Subsequently, RIP assays using anti-NMIIA also revealed the interaction between NMIIA and hsa_circ_0063865(Fig. 5C). Western blotting revealed that hsa_circ_0063865 overexpression increased NMIIA protein translation, rather than regulating NMIIA at the transcriptional level (Fig. 5D; Fig. S6G&H). More importantly, based on catRAPID predictions, we conducted electrophoretic mobility shift assay (EMSA) using two biotin-labeled fragments (probe1,126-177nt; probe2, 601-658nt) of hsa_circ_0063865 as probes, and confirmed that the 126-177nt region of hsa_circ_0063865 could bind to eEF1A2 (Fig. S6I), while 601-658nt region could bind to NMIIA (Fig. S6J). Thus, these results indicate that hsa_circ_0063865 functions as a modular scaffold, promoting NMIIA translation by facilitating the interaction between eEF1A2 and NMIIA.

NMIIA is encoded by MYH9, we used single-cell analysis to further investigate the characteristic functions of MYH9^+^ cancer cells (Fig. 5E). Here, a total of 4,328 differentially expressed (DE) genes were identified (Fig. 5F), and KEGG pathway analysis revealed that MYH9 (NMIIA) was closely associated with the actin filament organization, regulation of actin filament organization and cytoskeleton dependent cytokinesis (Fig. 5G). Additionally, GO analysis showed that the up-expressed MYH9 was correlated with cytoskeleton rearrangements (Fig. S6K). Further, using FITC-labeled ghost pen cyclic peptide staining, we discovered more obvious intermediate filaments and a more neatly arranged cytoskeleton in MYH9 silencing cells (Fig. 5H). Also, cells with lower hsa_circ_0063865 expression showed a more neatly arranged cytoskeleton compare with Si-NC cells (Fig. 5I). Cytoskeleton rearrangement is closely related to cell migration and invasion, we next investigate whether NMIIA participated in hsa_circ_0063865 induced migration and invasion, we interfered NMIIA in hsa_circ_0063865 overexpression cells. Transwell results demonstrated that NMIIA silencing abolished migration and invasion of Het-1A-T cells induced by hsa_circ_0063865 overexpression (Fig. 5J&K). In subcutaneous transplanted tumors derived from cells with hsa_circ_0063865 silencing, NMIIA expression was down-regulated (Fig. 5L). Conversely, in subcutaneous, liver metastasized, and lung metastasized tumors derived from hsa_circ_0063865 over-expressing cells, NMIIA expression was up-regulated (Fig. 5L-N). Similarly, IHC confirmed that NMIIA was significantly more highly expressed in cancer tissues than in adjacent normal tissues (Fig. S6L). In this section, we demonstrated that hsa_circ_0063865 acts as a molecular bridge to guide NMIIA protein translation, thereby promoting ESCC invasion and migration through cytoskeleton reorganization in vitro and in vivo.

### hsa_circ_0063865 inhibits endoplasmic reticulum stress in MYH9^+^ epithelial cell to mediate apoptosis via miR-450b-3p/RCN1 axis

**Fig. 6:**
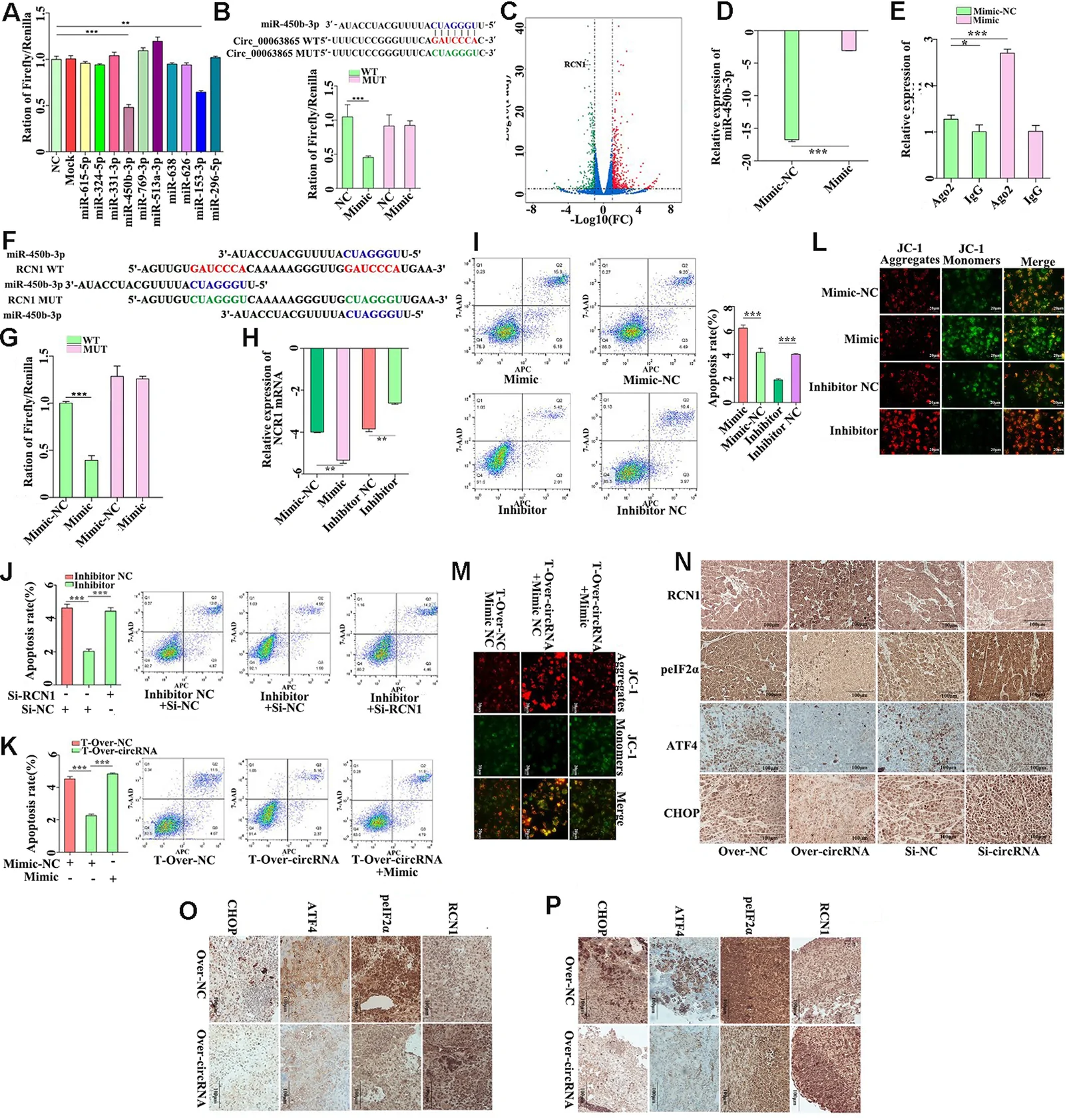
hsa_circ_0063865 inhibited endoplasmic reticulum stress of MYH9 + epithelial cell to mediate cell apoptosis via miR-450b-3p/RCN1 axis. **(A)** Screening of potential miRNAs of hsa_circ_0063865 detected by dual luciferase reporter assay. **(B)** Analysis of binding sites of hsa_circ_0063865 and miR-450b-3p using dual luciferase reporter assay. **(C)** Volcano plot of differentially expressed mRNAs. **(D)** Expressions of miR-450b-3p in cells transfected with miR-450b-3p mimic and mimic NC detected by qRT-PCR. **(E)** Anti-Ago2 RIP assay of combination of miR-450b-3p and RCN1 mRNA. **(F)** Sketch map of binding sites of hsa_circ_0063865 and miR-450b-3p. **(G)** Validation of binding sites of hsa_circ_0063865 and miR-450b-3p using dual luciferase reporter assay. **(H)** Analysis of effects on mRNA expression of RCN1 induced by over-expressing of under-expressing of miR-450b-3p. **(I)** Effects on cell apoptosis regulated by miR-450b-3p detected by flow cytometry using AnexinV-APC/7AAD. **(J) **RCN1 participated in regulating apoptosis induced by miR-450b-3p. **(K)** hsa_circ_0063865 inhibited cell apoptosis through regulating miR-450b-3p. **(L) **miR-450b-3p inhibited maintenance of mitochondrial membrane potential detected by JC-1 staining assay. **(M) **hsa_circ_0063865 promoted maintenance of mitochondrial membrane potential. **(N-P)** The levels of ER stress-related proteins in subcutaneous implanted tumors **(N)**, liver- metastasized tumors **(O)**, lung metastasized tumors **(P)** were detected by immunohistochemistry

The previous FISH assay indicated that hsa_circ_0063865 was mainly localized in the cytoplasm, here, we hypothesized that hsa_circ_0063865 may function as a miRNA sponge in ESCC. To identify the downstream miRNAs of hsa_circ_0063865, a total of 10 miRNAs were screened from the miRanda and circRNA interactome databases (Fig. 6A), and mutation assay verified that the bingding site of miR-450b-3p on hsa_circ_0063865 was GAUCCCA (Fig. 6B). Next, to further explore the mechanisms of miR-450b-3p in ESCC, RNA sequencing revealed that over-expression of miR-450b-3p significantly reduced RCN1 mRNA expression (Fig. 6C; Fig. S7A).Furthermore, anti-Ago2 immunoprecipitation combined with RT-qPCR showed miR-450b-3p overexpression promoted the enrichment of RCN1in the Ago2-immunoprecipitated complex compared with anti-IgG immunoprecipitates (Fig. 6D-E). In addition, a dual-luciferase reporter assay was performed by constructing WT and mutant (MUT) dual-luciferase reporter vectors of RCN1according to the potential binding sites with miR-450b-3p (Fig. 6F). The luciferase activity was significantly decreased by co-transfection of the WT reporter and miR-450b-3p mimics, but not the MUT reporter (Fig. 6G). RT-qPCR and Western blotting results demonstrated that miR-450b-3p overexpression decreased RCN1 protein and mRNA levels (Fig. 6H; Fig. S7B). In addition, the miR-450b-3p Mimic abolished the increase in RCN1 protein expression induced by hsa_circ_0063865 overexpression (Fig. S7C). Noteworthy, at single-cell resolution, we also found that RCN1 was mainly expressed MYH9^+^ epithelial cell subtypes (Fig. S7C&D) and Het-1A-T cells (Fig. 1K; Fig. S7F). These results suggest that hsa_circ_0063865 upregulates RCN1 expression by sponging miR-450b-3p in MYH9^+^ cancer cells.

Next, to further explore the potential functions of hsa_circ_0063865/miR-450b-3p/RCN1 axis, then we co-transfected hsa_circ_0063865 overexpression constructs with miR-450b-3p-mimic/inhibitor or RCN1 siRNA to conduct rescue experiment (Fig. S7G). Flow cytometry assay demonstrated that miR-450b-3p Mimic could promote cell apoptosis (Fig. 6I). Moreover, miR-450b-3p overexpression and RCN1 silencing could abolish the anti-apoptotic effect of hsa_circ_0063865 overexpression on MYH9^+^ epithelial cell (Fig. 6J&K). To further investigate the mechanisms by which miR-450b-3p/RCN1 regulated apoptosis, the single-cell data analysis that showed the ER stress-related genes DDIT3 and ATF4 were significantly enriched in MYH9-low cancer cells (Fig. S7H-J). Furthermore, western blot analysis demonstrated that miR-450b-3p Mimic could increase ER stress by up-regulating the expression of p-PERK, p-EIF2α, ATF4 and CHOP in MYH9^+^ cancer cells (Fig. S7K), which in turn increased mitochondrial membrane potential (Fig. 6L). However, opposite results were observed in cells with miR-450b-3p knockdown (Fig. 6L; Fig. S7K). Importantly, miR-450b-3p overexpression could abrogate the inhibitory effect of hsa_circ_0063865 on ER stress and mitochondrial membrane potential (Fig. 6M; Fig. S7L). Finally, using IHC analysis, we found that stable overexpression of hsa_circ_0063865 resulted in decreased expression of p-EIF2α, ATF4 and CHOP and increased expression of RCN1 in subcutaneous, liver metastasized, and lung metastasized tumors, the opposite result was observed in the hsa_circ_0063865 silencing group. Similarly, IHC confirmed that NMIIA was significantly more highly expressed in cancer tissues than in adjacent normal tissues (Fig. S7M). These findings suggested that the hsa_circ_0063865/miR-450b-3p/RCN1 axis slowed early apoptosis by inhibiting ER stress and increasing mitochondrial membrane potential, thereby accelerating the malignant transformation of Het-1A in vitro and in vivo.

## Discussion

EC is the sixth most common cancer worldwide and is associated with high malignancy and poor prognosis. In recent years, the incidence of EC has gradually increased. However, large geographical and temporal variations in incidence, even within small areas, suggesting the environment or exposure to certain risk factors are causally important. In China, for example, drinking tea at temperatures above 70℃ in Chao’shan [19] and smoking in Yan’ting [20] have been reported to significantly increase the risk of ESCC, respectively. However, in Huai’an, Jiangsu, another high-incidence area, we identified that nitrosamines DBP (e.g., NDMA and NMEA) and MC-LR were higher in drinking water than in other areas [4]. However, the precise relationship between NAs and MC-LR in carcinogenesis remains unclear. In this study, the median-effect principle showed that NAs and MC-LR exert both distinct and synergistic effects during chemical carcinogenesis, which was further confirmed by rat model of ESCC. Furthermore, we explored the potential mechanisms underlying this combined exposure and identified 292 “chemical-epithelial cell” related carcinogenic targets, which were primarily involved in MicroRNAs in cancer and cytoskeleton rearrangements. This finding is consistent with the report by Yang et al. and showed that MC-LR-mediated downregulation of miR-451a promotes liver cancer progression by inducing cytoskeletal damage [21]. Moreover, our previous study also confirmed that miR-21-5p and miR-218 are novel targets involved in NAs-induced esophageal carcinogenesis [22], suggesting that NAs or MC-LR may promote esophageal carcinogenesis by disrupting microRNAs and the cytoskeleton. However, the mechanisms underlying carcinogenesis induced by co-exposure remain unclear.Here, RNA-seq was performed to identify circRNAs in ESCC patients, and 571 differentially expressed circRNAs were identified in ESCC tissues, including 335 downregulated and 236 upregulated circRNAs. Among them, hsa_circ_0063865 exhibited the highest upregulation and was highly positively correlated with TNM stage. However, due to incomplete data, the association between hsa_circ_0063865 expression and overall survival, progression-free survival, and treatment response could not be determined. In addition, hsa_circ_0063865 was also highly expressed in NMBzA- and MC-LR-induced malignant transformed cells. Further, the result was confirmed by the loss- and gain-of-function experiments of hsa_circ_0063865 in Het-1 A cells and found hsa_circ_0063865 overexpression accelerated malignant transformation of Het-1 A cells. To date, however, no studies have reported the functions of hsa_circ_0063865. In the present study, we first demonstrated the effects of hsa_circ_0063865 on apoptosis, migration, and invasion, revealing the promoting role of hsa_circ_0063865 in malignant transformation. However, whether hsa_circ_0063865 exerts consistent effects in patient-derived esophageal cancer cell lines remains to be further validated. Further studies using hsa_circ_0063865 overexpression and knockdown models in cell lines such as Ec109 and KYSE450 are needed to validate its biological function. Even so, this result also suggests that hsa_circ_0063865 is a key target in NAs and MC-LR induced carcinogenesis and may be a potential biomarker for early ESCC diagnosis.

Notably, increasing evidence suggests that circRNAs can regulate the expressions and functions of combining proteins. Next, to screen for RNA-binding proteins interacted with hsa_circ_0063865, ChIRP-MS assay and RIP assays were performed, and we found that eEF1A2 interact with hsa_circ_0063865 in the cytoplasm. As a translation elongation factor, eEFlA2 directly binds aminoacyl tRNA and transports it to ribosomes, thereby promoting peptide elongation and ensuring the speed and accuracy of protein synthesis [23]. In recent years, eEFlA2 has also been reported as a proto-oncogene that promotes the progression of ovarian cancer and prostate cancer by regulating cytoskeleton remodeling, protein degradation, nuclear export and apoptosis [24], whereas it is not detected in normal visceral organs and tissues, including lung and pancreas. Most notably, the role of eEFlA2 in ESCC remains unknown. Considering its function in translation elongation, we screened the binding proteins of eEFlA2 by Co-IP-MS, identified and verified the interaction between eEF1A2 and NMIIA. Interestingly, eEF1A2 was more highly enriched with NMIIA in hsa_circ_0063865 overexpression cells, and hsa_circ_0063865 also interacted with NMIIA, suggesting that they may form a ternary complex with regulatory role. However, the interactions between them have not been deciphered. Recent reports have shown that some circRNAs may act as scaffolds to affect the interaction between two or more proteins. For instance, circ-CEA acts as a protein scaffold to enhance p53 phosphorylation at ser315 by promoting the interaction between p53 and CDK1 [25]. Liu et al. revealed that circ-DOPEY2 acts as a modular scaffold to mediate the ubiquitination and degradation of CPEB4 by enhancing combination of CPEB4 and TRIM25 [26]. Here, we found the hsa_circ_0063865 significantly increased NMIIA protein level, whereas the eEF1A2 expression did not change when hsa_circ_0063865 levels changed. In further study, we discovered siRNA-mediated eEF1A2 knockdown inhibited NMIIA translation, while eEF1A2 expression was not regulated by NMIIA. Therefore, we suggest that hsa_circ_0063865 acts as a molecular bridge to promote the NMIIA translation by guiding the interaction between eEF1A2 and NMIIA.

NMIIA, an actin-activated motor protein encoded by MYH9, is best known for its role in cell shape and movement, where it contributes to the regulation of cell migration, cell-substrate adhesion, and extracellular matrix remodeling [27]. However, NMIIA has tissue specific functions, and the role in tumors remain controversial. For instance, upregulation of NMIIA significantly enhanced cell proliferation and metastasis in lung, ovarian and pancreatic cancer [28, 29], but in squamous cell carcinomas of the head and neck, NMIIA acted as a tumor suppressor and inhibits tumor development and malignant progression [30]. Notably, higher expression of NMIIA has been identified in ESCC tissues with server lymphatic metastasis and poorer prognosis. Not surprisingly, in our present study, NMIIA expression was firstly detected in malignant transformed esophageal epithelial cells, and our results demonstrated a promoting effect of NMIIA on carcinogenesis, which was consistent with existing researches on ESCC [31, 32]. Currently, different mechanisms by which NMIIA promotes metastasis in tumors have mainly focused on epithelial-mesenchymal transition (EMT) process. Zhou et al. demonstrated that NMIIA regulates EMT via the Wnt/β-catenin signaling, which is critical for NMIIA-mediated invasion and metastasis in pancreatic cancer [33]. Another study in gastric cancer also indicated that silencing NMIIA represses metastasis by inhibiting EMT through suppression of MAPK/AKT signaling [34]. Similarly, we revealed that hsa_circ_0063865 also promotes the migration and invasion of Het-1 A-T cells by enhancing the translation of NMIIA in vitro and in vivo. However, the complex mechanism by which NMIIA mediates the EMT process in ESCC remains unclear. Recent studies have shown that cytoskeleton reorganization is indispensable in cell migration and invasion, which promoted EMT by enabling directional motility and dynamic cellular elongation. In addition, cytoskeletal reorganization increases membrane roughness and promotes pseudopodia formation, thereby enhancing the adhesion and migration ability of tumor cells [35]. It is known that cell cytoskeleton is mainly constituted by microfilaments, intermediate filaments and microtubules. As important cytoskeletal regulatory molecular motors, myosin IIs can form bipolar filaments that bind F-actin to exert cellular contractility [36]. In process of migration, myosin IIs provide the contractile and retractile forces required for rear retraction and forward movement of cells, and NMIIA contributes approximately 60% of the traction force [37]. It has been reported that ablation of NMIIA can significantly interfere with formation of stress fiber, mature of adhesion complex, disassembly of the adhesions, which then disrupted migration of colonic epithelial cells [38]. In nasopharyngeal carcinoma cells, deletion of NMIIA was also shown to inhibit both migration and invasion [39]. Thus, previous reports have demonstrated the importance and complexity of NMIIA in regulating metastasis by affecting cytoskeleton reorganization, although this may differ among tumor types. In the present study, we found the deletion of NMIIA inhibits cytoskeleton reorganization, whereas the opposite result was observed with hsa_circ_0063865 overexpression. One possible explanation is that NMIIA interacts with F-actin and contributes to actin contraction, thereby affecting cytoskeletal remodeling [40]. Although the mechanism by which NMIIA translocate to the cell membrane and binds to F-actin remains to be further investigated, our findings indicate that NMIIA may be involved in the malignant transformation of esophageal epithelial cells and may represent a potential target for preventing chemical carcinogenesis.

Although numerous ceRNA mechanisms have been elucidated in ESCC, such as circ_0021727/miR-23b-5p [41], circ_0007022/miR-338-3p [42] and circ-ATIC/miR-326 [43], the hsa_circ_0063865/miRNAs/mRNAs network has remained unclear. Therefore, dual-luciferase reporter assays and anti-Ago2 RIP assays were used in our study to prove the direct association between hsa_circ_0063865 and miR-450b-3p. Other studies have reported miR-450b-3p can inhibit proliferation of gastric cancer cell by inhibiting KLF7 [44], inhibit growth of hepatoma cell by inhibiting PGK1 [45], and suppress proliferation in breast cancer by targeting HER3 [46]. These results highlight an essential role of miR-450b-3p in cell-cycle regulation. However, in the case of chemical carcinogenic experiments, our studies found that low expression of miR-450b-3p significantly inhibited the apoptosis of Het-1 A-T cells, while rescue experiments showed that miR-450b-3p reversed the effect of hsa_circ_0063865 on apoptosis inhibition. Apoptosis is generally considered a mechanism for eliminating misplaced cells. However, in chemical carcinogenesis, uncontrolled cell apoptosis is a key mechanism in tumorigenesis. As described by Chen et al., 2,3,7,8-Tetrachlorodibenzo-p-dioxin (TCDD) increased DNA synthesis in keratinocytes and hepatocytes and was accompanied by an increased mutation rate [47]. As an anti-apoptosis agent, TCDD may allow genetically aberrant cells to evade apoptosis [48]. Similarly, we identified the mutation of the D16S539 and FGA sites in Het-1 A cells chronically exposed to NAs and MC-LR, while hsa_circ_0063865 overexpression mediated apoptosis inhibition may preferentially expand initiated cell populations over surrounding cells, thereby promoting malignant transformation. In conclusion, we demonstrated that MC-LR promoted NAs-induced esophagus tumorigenesis, at least in part, through inhibition of cellular apoptosis.

As a component of the ceRNAs regulatory network, miRNAs exert biofunction by targeting the 3ʹ-UTR of mRNA and reshaping gene expression. Further, target genes regulated by miR-450b-3p were screened by mRNA sequencing, which identified RCN1 as target gene of miR-450b-3p. As an EF-Hand calcium-binding proteins, RCN1 is located in ER cavity [49]. Some studies have revealed that RCN1 is aberrantly expressed in a variety of tumors, including prostate, kidney and oral cancer. Functionally, the RCN1 can inhibit cell apoptosis by inhibiting ERs in cancer cells. Xu et al., reported that RCN1 loss increased the UPR during drug-induced ER stress by activating PERK-CHOP signaling to induce apoptosis [49]. In prostate cancer, depletion of RCN1 triggers DU145 cell apoptosis in a caspase-dependent manner [50]. Here, our study is the first to report that miR-450b-3p mimic-mediated reduction of RCN1 inhibits ERs. ERs is triggered by three ER-resident transmembrane proteins, including ATF6, PERK, and IRE1α. It has been reported that PERK phosphorylates eIF2α, leading to ATF4 activation and subsequently increasing CHOP expression, which is linked to cell apoptosis [51]. In present study, following NAs and MC-LR exposure, hsa_circ_0063865 overexpression significantly inhibited the PERK/eIF2α/ATF4/CHOP pathway activation in vivo and in vitro, while the effect was reversed by transfection of miR-450b-3p mimics. However, when ERs is prolonged or severe, CHOP translation will be activated by ATF4, prolonged high expression of CHOP will then affect cell membrane potential and induce cell apoptosis. Notably, our present study demonstrated that the hsa_circ_0063865/miR-450b-3p/RCN1 axis preserves mitochondrial membrane potential by inhibiting ER stress, thereby reducing apoptosis. This is consistent with previous studies showing that PERK-eIF2α-ATF4-CHOP-mediated ERs promotes mitochondrial damage and induces apoptosis [52, 53]. Therefore, both apoptotic dysregulation and cytoskeletal reorganization are involved in chemical carcinogenesis. As reported by Zhang et al., BaP-induced upregulation of has_circ_0087385 promotes DNA damage in the early stage of chemical carcinogenesis and inhibits apoptosis in malignantly transformed cells [54]. In addition, cytoskeletal reorganization is a key process in tumor metastasis [55]. Wang et al. reported that miR-200b promotes RAC1activation, thereby enhancing the migration and invasion abilities of arsenic-transformed cells [56]. Taken together, these findings may indicate a temporal synergy between hsa_circ_0063865/miR-450b-3p/RCN1-mediated apoptosis and hsa_circ_0063865/eEF1A2/NMIIA-mediated cytoskeletal reorganization in chemical carcinogenesis. However, the detailed interaction mechanisms remain to be experimentally verified in future studies.

In conclusion, through an integrative analysis using a rat model of esophageal carcinogenesis, the median-effect principle, network toxicology and single-cell sequencing, we revealed a synergistic effect of NAs and MC-LR on chemical carcinogenesis and identified that 292 “chemical-epithelial cell” related carcinogenic targets primarily involved in MicroRNA in cancer and cytoskeleton rearrangements. Further, we identified that hsa_circ_0063865 as a chemical carcinogenesis-related circRNA that was upregulated in ESCC. Specifically, hsa_circ_0063865 acts as a molecular bridge to promote NMIIA translations by guiding combination of eEF1A2 and NMIIA, thereby enhancing cytoskeletal rearrangement and promoting malignant transformation of esophageal epithelial cells through increased migration and invasion. In addition, the hsa_circ_0063865/miR-450b-3p/RCN1 axis also accelerated malignant transformation by suppressing early apoptosis through i inhibition of ERs and preservation of mitochondrial membrane potential. Collectively, these findings provide new mechanistic insight into the initiation and progression of ESCC and suggest that hsa_circ_0063865 may be a potential biomarker associated with chemical carcinogenesis.

## Materials and methods

### Patients and samples

The study followed ethical principles in accordance with the Helsinki Declaration and approval was granted by the Ethics Committee of Southeast University-Aliated Zhongda Hospital (2018ZDKYSB016). A total of 110 pathologically diagnosed ESCC patients who had not received chemotherapy and/or radiotherapy were recruited at the Huai’an NO.1 People’s Hospital, Huai’an, Jiangsu, China, including 3 samples for CircRNA microarray analysis, 10 samples for single-cell RNA sequencing, and 97 samples for RT-qPCR validation. Surgically resected ESCC tissues and adjacent non-tumor tissues (5 cm away from the tumor edge) of patients who had signed written informed consents were collected for this research. All the samples were treated with RNA Locker reagent (Tiandz, Beijing, China) and then immediately frozen in liquid nitrogen. The characteristics of 110 patients were listed in Table S12. The study followed ethical principles in accordance with the Helsinki Declaration and approval was granted by the local university ethics committee.

### Single-cell RNA sequencing (scRNA-seq) analysis

To identify epithelial cell-specific differentially expressed genes in esophageal cancer, ten pairs of poorly differentiated ESCC tissues and their matched adjacent non-tumor tissues were collected for single-cell RNA sequencing using the DNBelab C4 platform (HaploX Biotechnology, Jiangxi, China). Briefly, single-cell suspensions were used for droplet generation, emulsion breakage, beads collection, reverse transcription, and cDNA amplification to generate barcoded libraries. Analysis of the scRNA-seq data was performed with Seurat package (v4.0.2). In quality control step, stringent filtering parameters were applied to weed out any empty oil beads, mortality, and doublet cells, excluding cells with fewer than 300 or more than 8,000 detected genes, or with mitochondrial content exceeding 10%. Then, the filtered scRNA-seq data were normalized by the “LogNormalize” method, and the hypervariable genes were identified by the “VST” method. Subsequently, the principal component analysis (PCA) was performed to identify the significantly principal components, the number of cell clusters were evaluated by the FindNeighbors and FindClusters function with a resolution of 1.0. Under the thresholds that *p* < 0.05 and |log2FoldChange (FC)| >1, the FindAllMarkers function in Seurat was performed to identify the marker genes. MYH9^high^ and MYH9^low^ cells were defined according to the median expression value as the threshold.

### Evaluation of LS-rHDLs based on the median-effect Principle

The median-effect analysis of Chou-Talalay [57] was employed to evaluate the combined effect of NAs and MC-LR, according to the reported method and parameter criteria [58]. Briefly, based on the dose ranges of NAs and MC-LR and the corresponding cell inhibition rates (Table S2), the combined parameters and graphs were analyzed by CompuSyn software program (version 3.0, ComboSyn Inc, Paramus, NJ). The combination index (CI) was calculated using the following equation: CI = D1/DX1 + D2/DX2 + αD1D2/DX1DX2, where D_x_ is the dose (NAs: DX1, MC-LR: DX2, or NAs + MC-LR: D1D2) required to achieve X% effect, For mutually exclusive drugs, α = 0; otherwise, α = 1. A CI value of < 1.0 indicates synergism, CI = 1.0 indicates an additive effect, and CI > 1.0 indicates antagonism.

### Rat model of ESCC

To assess the tumorigenic effect of NAs and MC-LR co-exposure, the F344-Rat model of ESCC were constructed based on previous experiments. Briefly, five-week-old male F344 rats were purchased from Siple-Bikai Laboratory Animal Co and raised under standard conditions. After acclimatization, the rats were randomly assigned into Control(*n* = 5), NMBzA(*n* = 10), MC-LR(*n* = 5) and NMBzA-MCLR(*n* = 8). Randomization was performed using a computer-generated random number table. NMBzA, MC-LR, and NMBzA-MCLR treated group were administered subcutaneous injection with 0.5 mg/kg NMBzA, 10 µg/kg MC-LR, and 0.5 mg/kg NMBzA+10 µg/kg MC-LR, respectively; The experiment was conducted three times per week for 5 weeks. After 31 weeks, the rats were euthanized and dissected, and all esophageal tissues were fixed with formalin and embedded for H&E staining. The sections were imaged digitally using the slide scanner at Pinuofei Biological Technology (Wuhan, China). To minimize bias, the histopathological analysis was performed by a pathologist blinded to the treatment groups. The animal experiments were approved by the Institutional Animal Care and Use Committee of Southeast University (20240301001). This project was executed in the Key Laboratory of Environment and Engineering of the Ministry of Education (Investigator:220222680).

### CircRNAs sequencing

To determine whether circRNAs are associated with ESCC progression, we performed circRNA sequencing analysis of human esophageal samples from three patients. Briefly, the RNA was isolated from 3 patients, and liner RNAs were removed by Ribonuclease R (Epicentre, USA). The circular RNA library was then detected using Illumina HiSeq 4000 system (Illumia, USA), the capacity of raw reads for each sample should be no less than 8 Gigabytes. Differentially expressed circRNAs were identified based on a fold change ≥ 2 and a P-value < 0.05 in comparisons between tumoral tissue or non-tumoral tissue. The detailed information is shown in Table S13.

### DNA isolation, PCR amplification and sanger sequencing

DP304 TIANamp Genomic DNA Kit was used for extracting genomic DNA from tissues and cell lines, and then amplified cDNA or/and genomic DNA by LA Taq^®^ polymerase (Takara, Japan). The products of PCR amplification were isolated and identified by agarose gel electrophoresis on a 3% agarose gel at 90 V for 30 min. The Sanger sequencing was used to detect the entire length of the amplification products by Divergent primer. The back-splice junction of hsa_circ_0063865 was identified by ABI 3730 Sanger sequencing (Sangon Biotech, Shanghai, China).

### RNA isolation and quantitative real-time PCR (RT-qPCR)

NE-PER™ Nuclear and Cytoplasmic Extraction Reagents (Thermo, USA) were used to isolate and extract RNAs from nucleus and cytoplasm for detecting the expression level of hsa_circ_0063865. Total RNAs in tissues and cell lines were extracted using TRIzol reagent (Invitrogen, USA), cDNAs were then synthesized using M-MLV Reverse transcriptase (Invitrogen, USA). The RT-qPCR was performed using SYBR^®^ Green Realtime PCR Master Mix -Plus- (Toyobo, Japan). GAPDH was utilized as internal control for circRNAs and mRNAs. Transcription levels were presented as fold changes using the ΔCT or 2^−ΔΔCT^ methods. The sequences of primers were shown in Table S14.

### Cell transfection

The sh-hsa_circ_0063865 and full-length sequence of hsa_circ_0063865 were synthesized and inserted into the pHBLV-U6-Puro-RFP and pLent-EF1a-circRNA-CMV-RFP-P2A-Puro vector for stable overexpression and knockdown, respectively. siRNAs specifically against eEF1A2 and NMIIA were synthesized by RiboBio Biotech (Guangzhou, China), and transfected into cells using Lipofectamine^®^ 3000 transfection reagent (Invitrogen, USA). The malignant transformed Het-1A(Het-1A-T) was induced by exposing of 100 nmol/L NMBzA (Mergene, China) and 100 nmol/L MC-LR (Alexis Biochemical, Switzerland) for 45 generations. The Het-1A cells transfected with sh-hsa_circ_0063865 and over-hsa_circ_0063865 were exposed to NMBzA and MC-LR for 45 generations to induce malignant transformation. All cells were cultured in DMEM medium (Gibico, USA) supplemented with 10% fetal bovine serum (FBS, HyClone, USA), 1% penicillin-streptomycin (HyClone, USA) at 37 °C with 5% CO_2_. Subsequently, Plate Cloning Assay(1 × 10^2^ cells/well to 6-well plates, Jet Biofil, Guangzhou, China) and Soft-agar colony forming assay (8 × 10^2^ cells/well to 6-well plates, Jet Biofil, Guangzhou, China) were performed for analysing degrees of malignant transformation. The shRNA/siRNA sequences are listed in Table S15.

### Experimental in vivo metastasis models

A total of 24 male BALB/c nude mice (aged 4 weeks, Vital River Laboratory Animal Technology, Beijing, China) were injected intravenously through the tail vein (2 × 10^6^ cells per mouse) with Het-1A cells which (Over-NC, Over-circRNA, Si-NC, Si-circRNA) cultured in medium containing NMBzA and MC-LR for 40 generations. Before injection, Het-1A cells were transfected with PLVX-Luc2-Blast for expressing luciferase. When raised for 6 weeks, IVIS Lumina XRMS Series III system (PerkinElmer, USA) was used for detecting metastasized tumors, 15 min before detection, 10µL (15 mg/mL) D-Luciferin (BioVision, USA) were intraperitoneally injected into each mouse. Dissect liver and lung tissues, make the tissues into homogenate, LB942 microplate multifunction analyzer (Berthold, Germany) was then used for detecting luciferase in homogenate. H&E staining was performed for analyzing morphologies of tumor cells.

### Co-IP/MS assay

Pierce™ Crosslink Magnetic IP/Co-IP Kit was used for screening interacting proteins of eEF1A2 using anti-eEF1A2 (Abcam, USA). Briefly, the total proteins from cells were extracted using IP lysis buffer. After BCA protein quantification, the protein lysates were incubated overnight with anti-eEF1A2 antibody (Abcam, USA) or anti-IgG antibody (Abcam, USA) at 4 °C. Then, the immune complexes were captured by Protein A/G Magnetic Beads. The protein was separated by SDS-PAGE and isualized by silver staining. Finally, the differential bands were excised and identified using EASY-nLC 1200TM UPLC system and Q Exactive Mass Spectrometer (Thermo, USA). Proteins eluted from the protein A/G beads were detected by western blot.

### Fluorescent in situ hybridization (FISH) assay

The FISH assay was used to detect the abundance and location of hsa_circ_0063865 in cells according to the manufacturer’s instructions of Fluorescent in Situ Hybridization Kit (RiboBio, China). Briefly, Het-1A cells were seeded in confocal dishes and fixed in 4% formaldehyde for 10 min at 37 °C. After that, the cells were incubated with Cy3 labeled FISH probe specifically against back-splicing junction of hsa_circ_0063865, U6 probe and 18S probe (RiboBio, China). U6 and 18S were selected as internal control for indicating nucleus and cytoplasm, DAPI was used for labeling nucleus. Finally, the images were captured with FV1000 laser confocal microscope (Olympus, Tokyo, Japan).

### RIP assay

RIP assay was performed for identifying combinations of protein and RNA using Magna RIP™ RNA-Binding Protein Immunoprecipitation Kit according to the manufacturer’s instructions. Briefly, approximately 1 × 10^7^ hsa_circ_0063865-overexpressing Het-1A-T cells were lysed with 100µL RIP lysis buffer supplemented with 0.5µL protease inhibitor cocktail and 0.5µL RNase inhibitor. Magnetic beads were incubated with 5 µg of antibodies against NMIIA, eEF1A2 and normal IgG overnight at 4 °C. After immunoprecipitation, the coprecipitated RNAs were extracted and purified using the phenol-chloroform method. The fold enrichment of hsa_circ_0063865 was detected by RT-qPCR.

### ChromatinIsolation by RNA purification (ChIRP) and mass spectrometry assay

Biotin labeled ChIRP probes specifically against back-splicing junction of hsa_circ_0063865 (5′-/5bio/-CTGCCTGAGCCGCTGTCTTT-3′)were designed and synthesized by RiboBio Biotech Co., Ltd (Guangzhou, China), the complementary sequences against probes were used as NC probes(5′-/5bio/-GACGGACTCGGCGACAGAAA-3′). Briefly, approximately 2 × 10^7^ hsa_circ_0063865-overexpressing Het-1A-T cells were resuspended with prechilled PBS, and then crosslinked with 3% formaldehyde. After termination of crosslinking by adding 1.25 M glycine, the cells were sonicated in lysis buffer. Subsequently, the cell lysate was incubated with biotin-labeled probes of the control and hsa_circ_0063865, and then C-1 Magnetic beads (Invitrogen, USA) was used for extracting proteins binding with hsa_circ_0063865. Finally, the binding proteins were then separated by SDS-polyacrylamide electrophoresis and stained by silver staining using RapidSilver Stain Kit (Beyotime, China). EASY-nLC 1200™ UPLC system and Q Exactive Mass Spectrometer (Thermo, USA) were used for identifying binding proteins separated by ChIRP assay. eEF1A2A and Nucleophosmin(NPM) were identified with false discovery rate ≤ 70% and unique peptide ≥ 2. Subsequently, the combinations of hsa_circ_0063865 and identified eEF1A2 and NPM were verified by RIP assay using Anti-eEF1A2 antibody (Abcam, USA) and Anti- NPM antibody (Abcam, USA).

### EMSA

The biotin-labeled probes (probe1 and probe 2) were synthetized from Generay Biotech Co.,Ltd (Shanghai, China). EMSA was performed using the Chemiluminescent RNA EMSA Kit (Beyotime, China) in accordance with the manufacturer’s protocols. Briefly, the cell lysates, probe1 or probe 2, anti-eEF1A2 or anti-NMIIA, and REMSA Binding Buffer were incubated on ice for 20 min. The mixture was separated using 8% polyacrylamide gels and transferred onto 0.45 μm nylon membrane (Solarbio, China). After cross-linked by ultraviolet light, the bands were detected by Chemiluminescence Imaging.

### Dual-luciferase reporter assay

Fragment of full length of hsa_circ_0063865 was inserted into pmirGLO Dual-Luciferase Reporter Vector (Promega, USA) for screening bindings miRNAs of hsa_circ_0063865 from candidate miRNAs. MiRNA mimics (RiboBio, China) were then co-transfected with the recombinant Dual-Luciferase Reporter Vector, mimic NC was selected as internal control, LB942 microplate multifunction analyzer (Berthold, Germany) was used for detecting luciferases using Dual-Luciferase Reporter Assay System (Promega, USA). Binding sites of miR-450b-3p with hsa_circ_0063865 were then verified by constructing recombinant Dual-Luciferase Reporter Vector containing mutated binding sites of miR-450b-3p. Fragments of full-length of RCN1 3′untranslated region (3′UTR) containing wild-type (WT) or mutated (MUT) binding sites of miR-450b-3p were inserted into Dual-Luciferase Reporter Vector for identifying the combination of miR-450b-3p and RCN1.

### Analysis of cell proliferation, invasion, migration ability, and apoptosis

For EdU (5-ethynyl-2′-deoxyuridine) incorporation assay, the Het-1A cells (Over-NC, Over-circRNA, Si-NC, Si-circRNA) cultured in medium containing NMBzA and MC-LR for 40 generations were seeded in 96-well plates at the density of 1 × 10^4^/well. After 24 h, the cells were incubated with the EdU working solution for 2 h at 37℃ in the darkness. Finally, Hoechst 3342 was used for staining nucleus and photographed with an inverted fluorescence microscope with excitation at 519 and 460 nm. For cell migration assays, after different treatments, cells were planted at the concentration of 5 × 10^4^/champer and cultured with complete medium for 24 h. For the cell invasion assays, 5 × 10^5^ cells were plated into the Transwell insert coated with Matrigel (Corning, USA) and cultured with complete medium for 24 h. Finally, all cells were fixed with 95% ethanol and stained with 0.1% crystal violet dye. For detection of apoptosis, cells were stained with annexin V-FITC/PI at RT in the dark. The rate of apoptosis was analyzed by flow cytometry. All assays were independently performed in triplicate.

### JC-1 staining assay

Mitochondrial Membrane Potential Detection Kit (Beyotime, China) was used for detecting Mitochondrial membrane potential according to the manufacturer’s instructions. In brief, cells were seeded in 6-well plates at the density of 3 × 10^5^/well and transfected with miR-450b-3p mimic/Inhibitor. After 48 h, cells were stained with the JC-1 staining solution (5µg/mL) for 15 min at 37 C in the darkness and observed under inverted fluorescence microscope.

### Phalloidin staining

To observe the cytoskeletal morphology, F-actin were stained using Actin-Tracker Green (Beyotime, China) according to the manufacturer’s instructions. Briefly, cells were fixed using 3.7% formaldehyde solution in PBS for 10 min, then 0.1% Triton-X was used for permeabilizing cell membrane for 10 min at RT. After washing twice with PBS, cells were incubated with Actin-Tracker Green (1:50 dilution) for 1 h at RT, then wash cells for three times using 0.1% Triton-X in PBS. Finally, the Axio Vert.A1 fluorescence microscope was used for obtaining fluorescent images of F-actin.

### Western blot assay

The total protein was extracted from the cells using RIPA Lysis Buffer (Beyotime, China) containing 2% Phosphatase inhibitor Cocktail Set V (Millipore, USA) and 0.5% 200 mM PMSF (CST, USA). Protein concentrations were detected using PierceTM BCA Protein Assay Kit (Thermo, USA). A total of 30 µg of protein was separated using sodium SDS-PAGE (Epizyme, China) and transferred onto PVDF membranes (Millipore, USA). 5% w/v nonfat milk was used for blocking proteins on PVDF membranes for 1 h at RT, then incubate the membranes with specific primary antibodies overnight at 4 °C, HRP-conjugated secondary antibodies were then incubated for 1 h at RT. The signals were detected using a Super ECL Plus Kit (Thermo, USA) and determined by quantitative analysis using Tanon-5200 Chemiluminescence gel imaging system (Tanon, China). The information of antibodies was listed in Supplementary Table 16.

### Immunohistochemically staining (IHC) assay

The nude mice xenograft tissues (xenograft tumor, liver and lung) were fixed with 4% paraformaldehyde for 24 h and then embedded in paraffin for making slices with a thickness of 4 μm. After deparaffinization and rehydration, the tissues were then blocked with endogenous peroxidase for 15 min, then incubate tissues with TBST containing 0.5% primary antibody (PCNA, E-cadherin, NMIIA, eEF1A2, CHOP, pPERK, peIF2α and RCN1) at 4 °C overnight. Subsequently, samples were incubated with an HRP-IgG secondary antibody at RT for 1 h and then incubated with streptavidin peroxidase complex. Finally, samples were stained by diaminobenzidine and counterstained with hematoxylin and analyzed with a light microscope. The information of antibodies was listed in Table S16.

### Statistical analysis

All statistical data were expressed as the mean ± Standard Deviation (‾x ± SD) and representative of at least three independent experiments. Two-tailed Student’s t test was used for comparisons between two groups. One-way analysis of variance (ANOVA) was used for multi-group comparisons followed by Bonferroni post-hoc analysis. Categorical data were analyzed using Fisher’s exact test χ2-test, as appropriate. Statistical analyses were performed using SPSS 16.0 software and Prism 7.04 software. *P* value of < 0.05 was calculated as statistically significant.

## Supplementary Information

Below is the link to the electronic supplementary material.

Supplementary Material 1

Supplementary Material 2

## Data Availability

All data generated or analyzed during this study are included either in this article or in the supplementary information files.
