# Supplementary material for: hsa_circ_0063865 regulates cytoskeleton rearrangement and endoplasmic reticulum stress to promote esophageal tumorigenesis under co-exposure to N-nitrosamines and microcystin
Source: J Transl Med. 2026 Jul 7;24:1228. doi: 10.1186/s12967-026-08554-8 (PMC13628824; doi:10.1186/s12967-026-08554-8)
Supplement: Supplementary file 1 [file 12967_2026_8554_MOESM1_ESM.doc]

**Figure S1**

Figure S1 Single-cell data quality control(A) and marker genes(B)

**Figure S2**

**Fig.S2 NAs combined with MC-LR induced esophageal carcinogenesis by regulating cytoskeletal rearrangement and endoplasmic reticulum stress of epithelial cell subsets**

（A）Comparative STR sequence analysis of Het-1A and Het-1A-T. (B) Soft agar clone formed by Het-1A-T.

**Figure S3**

**Fig.S3** **Chemical carcinogenesis-related circ_0063865 is upregulated in ESCC**

(A) Category distribution of identified circRNAs. (B) Venn analysis of identified circRNAs and circBase database. (C) Distribution of circRNAs on Chromosomes. (D) Density of circRNAs on Chromosomes. (E) Length distribution of identified circRNAs. (F) Exon distribution of circRNAs. (G) Expressive circos diagram of circRNAs identified by RNA-sequencing.(H) ROC curve was conducted to evaluate the potential diagnostic value of hsa_circ_0063865 in ESCC. (I)The expression level of hsa_circ_0063865 in Het-1A cell with RNase R treatment.

**Figure S4**

**Fig.S4** **Circ_0063865 is involved in NAs and MC-LR co-exposure-induced malignant transformation of human esophageal epithelial cells**

(A) Detection of overexpressing efficiency of hsa_circ_0063865 in Het-1A. (B) Detection of interfering efficiency of hsa_circ_0063865 in Het-1A. (C) Effects analysis of over-expressing hsa_circ_0063865 on subcutaneous tumorigenesis in nude mice. (D) Effects analysis of under-expressing hsa_circ_0063865 on subcutaneous tumorigenesis in nude mice. (E) Pictures of nude mice raised for 4 weeks after injection of Het-1A-T.

**Figure S5**

**Fig.S5** **Regulatory function of circ_0063865 in NAs combined with MC-LR-induced carcinogenesis**

(A) Detection of overexpressing efficiency of hsa_circ_0063865 in Het-1A-T. (B) Detection of interfering efficiency of hsa_circ_0063865 in Het-1A-T.

**Figure S6**

**Fig.S6 Circ_0063865 driver cytoskeleton rearrangements of MYH9^+^ epithelial cell by enhancing** **the stability of the eEF1A2-****NMIIA complex**

(A)Silver staining assay of hsa_circ_0063865 binding proteins pulled by CHIRP probes. (B) Total ion current analysis of eEF1A2-binding proteins separated by Co-IP. (C) Product ion mass spectrum of eEF1A2. (D) Combination of eEF1A2 and hsa_circ_0063865 predicted by catRAPID. (E) Silver staining assay of eEF1A2-binding proteins separated by Co-IP. (F) Product ion mass spectrum of NMIIA.(G)hsa_circ_0063865 promoted expression of NMIIA detected by WB. (H) Down-regulation of eEF1A2 inhibited expression of NMIIA. (I-J) RNA-EMSA assay confirmed the interaction of hsa_circ_0063865(Probe1:126-177bp) with eEF1A2(I) and hsa_circ_0063865 (601-658bp) with NMIIA (J) (purple: Free probe; Red: Super-shift; Blue: Shift).(K)GO enrichment analysis of differentially expressed genes.(L) IHC analysis of NMIIA expression in patient tissue samples

**Figure S7**

**Fig.S7** **Circ_0063865 inhibited endoplasmic reticulum stress of MYH9+ epithelial cell to mediate cell apoptosis via miR-450b-3p/RCN1 axis**

(A)Cluster analysis of differentially expressed mRNAs between cells transfected with hsa-miR-450b-3p mimic and mimic NC. (B) Analysis of effects on protein expression of RCN1 induced by over-expressing of under-expressing of miR-450b-3p. (C)hsa_circ_0063865 promoted expression of RCN1 through regulating hsa_circ_0063865. (D)The violin plot of RCN1 expression in MYH9high and MYH9Low cells. (E) Comparative analysis of mRNA expressions of RCN1 in Het-1A and Het-1A-T. (F)Violin plot of RCN1expression in MYH9high and MYH9Low cells.(G)Interfering efficiency analysis of si-RCN1 detected by Western blot. (H)Bubble Chart of DDIT3(CHOP) and ATF4 expression in MYH9high and MYH9Low cells. (I)The violin plot of DDIT3(CHOP) expression in MYH9high and MYH9Low cells. (J)The violin plot of ATF4 expression in MYH9high and MYH9Low cells. (K)miR-450b-3p activated endoplasmic reticulum stress pathway. (L) hsa_circ_0063865 inhibited endoplasmic reticulum stress pathway through regulating RCN1. (M) IHC analysis of RCN1 expression in patient tissue samples
