# Supplementary material for: hsa_circ_0063865 regulates cytoskeleton rearrangement and endoplasmic reticulum stress to promote esophageal tumorigenesis under co-exposure to N-nitrosamines and microcystin
Source: J Transl Med. 2026 Jul 7;24:1228. doi: 10.1186/s12967-026-08554-8 (PMC13628824; doi:10.1186/s12967-026-08554-8)
Supplement: Supplementary file 2 [file 12967_2026_8554_MOESM2_ESM.doc]

**Supplementary Table 1. Comparison of the incidence among the four treatment groups using Fisher-Freeman-Halton**

| Group | Incidence n (%) | No incidence n (%) | Total |
| --- | --- | --- | --- |
| Blank | 0 | 5 | 5 |
| NMBzA-MC-LR | 8 | 0 | 8 |
| NMBzA | 6 | 4 | 10 |
| MC-LR | 0 | 5 | 5 |
| Fisher-Freeman-Halton | P<0.001 | | |

**Supplementary Table 2. Growth Inhibition Rate of Het-1A Cells (%；n=3 ；x̄ ± SD)**

| NMBzA |  | MC-LR | | | | |
| --- | --- | --- | --- | --- | --- | --- |
|  | 0 | 1 nmol/L | 10 nmol/L | 100 nmol/L | 1 μmol/L | 10 μmol/L |
| 0 | 0 | 15.62±1.7%* | 10.44±1.2% | 18.91±2.3% | 25.59±4.2%* | 36.48±2.7%* |
| 1 nmol/L | 13.16±1.3% | 8.71±1.1% | 3.41±2.1%* | 24.33±0.8% | 29.17±1.5%* | 37.62±2.5%* |
| 10 nmol/L | 17.72±1.0% | 9.61±2.1%* | 6.1±7.3%* | 34.41±1.9%* | 35.77±6.7%* | 39.53±1.3%* |
| 100 nmol/L | 21.19±3.3%* | 23.53±0.8%* | 21.35±3.2%* | 36.75±1.7%* | 46.8±1.6%* | 47.65±3.9%* |
| 1 μmol/L | 33.44±2.7%* | 30.29±2.2%* | 27.84±5.4%* | 45.55±2.7%* | 46.32±4.6%* | 51.19±1.7%* |
| 10 μmol/L | 35.59±1.8%* | 33.5±3.1%* | 32.52±3.5%* | 46.69±10.4%* | 46.07±1.3%* | 57.41±3.5%* |
| 100 μmol/L | 45.43±5.3%* | 45.57±2.2%* | 35.06±2.3%* | 52.64±6.7%* | 56.27±4.5%* | 62.25±6.4%* |
| 1 mmol/L | 48.28±4.5%* | 50.11±8.6%* | 36.48±2.2% | 55.34±9.6%* | 58.82±5.6% | 64.63±8.3%* |

*P<0.05

**Supplementary Table 3. Analysis of Variance for Factorial Design**

| Parameters | Sum of Squares | df | Mean Square | F | P |
| --- | --- | --- | --- | --- | --- |
| MC-LR | 115.4 | 4 | 28.9 | 1312.2 | <0.05 |
| NMBzA | 133.2 | 6 | 22.2 | 5144.5 | <0.05 |
| MC-LR+NMBzA | 24.5 | 24 | 1.02 | 522.5 | <0.05 |
| Error | 3.4 | 70 | 0.05 |  |  |
| Total | 276.5 | 104 |  |  |  |

**Supplementary Table 4. Combination index(CI)of combined treatment of NMBzA and MC-LR against Het-1A cells**

| Toxicant | Slope(m) | Median-effect dose (Dm,μmol/L) | Correlation coefficient(r) |
| --- | --- | --- | --- |
| MC-LR | 0.03285 | 1329.086 | 0.87231 |
| NMBzA | 0.14887 | 323.619 | 0.98067 |
| NMBzA+MC-LR | 0.18107 | 5.26 | 0.87908 |

**Supplementary Table 5.** **Combination index(CI)of combined treatment of NMBzA and MC-LR against Het-1A cells**

| NMBzA |  | MC-LR | | | | |
| --- | --- | --- | --- | --- | --- | --- |
|  | 0 | 1 nmol/L | 10 nmol/L | 100 nmol/L | 1 μmol/L | 10 μmol/L |
| 0 | - | - | - | - | - | - |
| 1 nmol/L | - | - | - | 0.1881 | 0.3315 | 0.2394 |
| 10 nmol/L | - | - | - | 0.0079 | 0.0425 | 0.1378 |
| 100 nmol/L | - | 0.7849 | 2.1102 | 0.0108 | 0.0022 | 0.0144 |
| 1 μmol/L | - | 0.6373 | 1.5231 | 0.0057 | 0.0064 | 0.0064 |
| 10 μmol/L | - | 2.1768 | 3.0074 | 0.0394 | 0.0493 | 0.0027 |
| 100 μmol/L | - | 0.5442 | 13.1709 | 0.0696 | 0.0241 | 0.0042 |
| 1 mmol/L | - | 1.4508 | 84.0606 | 0.3157 | 0.1126 | 0.0189 |

Note: “-” indicates the single-agent control point or outlier.

**Supplementary Table 6. Fraction Affected (fa) of combined treatment of NMBzA and MC-LR against Het-1A cells**

| NMBzA |  | MC-LR | | | | |
| --- | --- | --- | --- | --- | --- | --- |
|  | 0 | 1 nmol/L | 10 nmol/L | 100 nmol/L | 1 μmol/L | 10 μmol/L |
| 0 | - | - | - | - | - | - |
| 1 nmol/L | - | **0.0871** | 0.0341 | 0.2433 | 0.2917 | 0.3762 |
| 10 nmol/L | - | 0.0961 | **0.0610** | 0.3441 | 0.3577 | 0.3953 |
| 100 nmol/L | - | 0.2353 | 0.2135 | **0.3675** | 0.4680 | 0.4765 |
| 1 μmol/L | - | 0.3029 | 0.2784 | 0.4555 | **0.4632** | 0.5119 |
| 10 μmol/L | - | 0.3350 | 0.3252 | 0.4669 | 0.4607 | **0.5741** |
| 100 μmol/L | - | 0.4557 | 0.3506 | 0.5264 | 0.5627 | 0.6225 |
| 1 mmol/L | - | 0.5011 | 0.3648 | 0.5534 | 0.5882 | 0.6463 |

Note: “-” indicates the single-agent control point

**Supplementary Table 7. Combination index(CI)of combined treatment of NMBzA and MC-LR against Het-1A cells**

| Fa | 0.1 | 0.2 | 0.3 | 0.4 | 0.5 | 0.6 | 0.7 | 0.8 | 0.9 |
| --- | --- | --- | --- | --- | --- | --- | --- | --- | --- |
| CI | 62.86 | 2.50 | 0.29 | 0.05 | 0.01 | 0.002 | 3.49× 10^^-4^ | 4.12× 10^^-5^ | 1.65× 10^^-5^ |

**Supplementary Table 8. Cell cycle of Het-1A Cells（P20;n=3;x̄ ± S）**

| Group | Cell cycle | F | P |
| --- | --- | --- | --- |
| G1 |  |  |  |
| Control | 62.94±0.25 |  |  |
| MC-LR | 30.85±3.34 | 234.968 | <0.05 |
| NMBzA | 27.74±1.40 | 429.125 | <0.05 |
| NMBzA+ MC-LR | 42.13±3.54 | 1620.293 | <0.05 |
| S |  |  |  |
| Control | 24.80±0.50 |  |  |
| MC-LR | 37.67±1.81 | 4.037 | 0.079 |
| NMBzA | 56.20±1.25 | 905.151 | <0.05 |
| NMBzA+ MC-LR | 41.01±1.66 | 590.523 | <0.05 |
| G2 |  |  |  |
| Control | 12.26±0.68 |  |  |
| MC-LR | 31.48±1.88 | 300.901 | <0.05 |
| NMBzA | 16.05±0.87 | 87.967 | <0.05 |
| NMBzA+ MC-LR | 16.86±1.94 | 245.196 | <0.05 |

**Supplementary Table 9. Migration and invasion of Het-1A Cells（P20;n=3;x̄ ± S）**

| Group | Number of cells | F | P |
| --- | --- | --- | --- |
| Cell migration |  |  |  |
| Control | 5.67±0.34 |  |  |
| MC-LR | 6.06±0.42 | 3.091 | 0.117 |
| NMBzA | 12.03±0.61 | 146.371 | <0.05 |
| NMBzA+ MC-LR | 13.67±2.46 | 6.172 | <0.05 |
| Cell invasion |  |  |  |
| Control | 8.67±1.34 |  |  |
| MC-LR | 15.06±2.42 | 108.541 | <0.05 |
| NMBzA | 19.03±2.61 | 299.101 | <0.05 |
| NMBzA+ MC-LR | 24.67±3.46 | 10.422 | <0.05 |

**Supplementary Table 10. Differently expressed circRNAs between ESCC tissues and normal non-tumor tissues (Top 20 upregulated and downregulated)**

| Name | Base mean | Fold change | Dysregulation | Chromosome | circRNA Length | Type | Host gene | ***P*** value |
| --- | --- | --- | --- | --- | --- | --- | --- | --- |
| Hsa_circ_0063865 | 13.63 | 43.63 | Up | chr22 | 1007 | Intergenic | - | ＜0.001 |
| Hsa_circ_0008796 | 6.52 | 25.02 | Up | chr9 | 50614 | Intergenic | - | ＜0.001 |
| circRNA_19252 | 7.42 | 25.01 | Up | chr2 | 701 | Intergenic | - | ＜0.001 |
| circRNA_11038 | 6.74 | 24.19 | Up | chr14 | 2996 | Sense-overlapping | CEP128 | ＜0.001 |
| circRNA_25261 | 6.48 | 23.86 | Up | chr3 | 20510 | Sense-overlapping | FNDC3B | ＜0.001 |
| circRNA_06726 | 6.89 | 23.44 | Up | chr11 | 275 | Sense-overlapping | MMP12 | ＜0.001 |
| circRNA_34471 | 6.23 | 18.41 | Up | chr8 | 331 | Sense-overlapping | RIMS2 | 0.002 |
| Hsa_circ_0002557 | 4.86 | 17.50 | Up | chr13 | 194 | Exonic | FARP1 | 0.002 |
| Hsa_circ_0079227 | 5.03 | 17.27 | Up | chr7 | 186 | Exonic | FOXK1 | 0.002 |
| circRNA_37055 | 4.58 | 15.56 | Up | chrX | 4304 | Intergenic | - | 0.003 |
| Hsa_circ_0060849 | 4.39 | 15.41 | Up | chr20 | 1202 | Sense-overlapping | NFATC2 | 0.004 |
| circRNA_07175 | 5.29 | 15.23 | Up | chr12 | 313 | Intergenic | - | 0.004 |
| circRNA_19586 | 5.84 | 15.21 | Up | chr2 | 537 | Sense-overlapping | PTPN4 | 0.004 |
| Hsa_circ_0005114 | 4.62 | 15.01 | Up | chr8 | 768 | Sense-overlapping | RIMS2 | 0.004 |
| circRNA_20896 | 4.16 | 14.95 | Up | chr2 | 530 | Sense-overlapping | SP140L | 0.004 |
| circRNA_19020 | 4.33 | 14.82 | Up | chr2 | 252 | Exonic | WDPCP | 0.004 |
| Hsa_circ_0001707 | 23.26 | 14.81 | Up | chr7 | 427 | Intronic | ABCA13 | ＜0.001 |
| circRNA_02464 | 4.31 | 14.71 | Up | chr1 | 929 | Intergenic | - | 0.004 |
| Hsa_circ_0006332 | 4.39 | 14.60 | Up | chr20 | 554 | Sense-overlapping | MYBL2 | 0.004 |
| circRNA_09450 | 4.23 | 14.44 | Up | chr13 | 326 | Sense-overlapping | EPSTI1 | 0.005 |
| circRNA_12733 | 20.07 | 77.04 | Down | chr15 | 338 | Sense-overlapping | RHCG | ＜0.001 |
| circRNA_26487 | 9.54 | 35.56 | Down | chr4 | 780 | Sense-overlapping | TMPRSS11B | ＜0.001 |
| circRNA_29087 | 9.52 | 34.21 | Down | chr5 | 195 | Sense-overlapping | SPINK5 | ＜0.001 |
| circRNA_26485 | 8.40 | 31.29 | Down | chr4 | 829 | Sense-overlapping | TMPRSS11B | ＜0.001 |
| circRNA_04922 | 8.27 | 30.81 | Down | chr10 | 330 | Sense-overlapping | CYP2C19 | ＜0.001 |
| circRNA_15794 | 8.15 | 30.51 | Down | chr17 | 2198 | Intergenic | - | ＜0.001 |
| circRNA_26365 | 7.74 | 27.71 | Down | chr4 | 473 | Sense-overlapping | CWH43 | ＜0.001 |
| Hsa_circ_0052577 | 6.94 | 25.66 | Down | chr2 | 991 | Sense-overlapping | GRHL1 | ＜0.001 |
| Hsa_circ_0043462 | 14.03 | 24.95 | Down | chr17 | 327 | Sense-overlapping | ERBB2 | ＜0.001 |
| circRNA_14632 | 6.73 | 24.32 | Down | chr17 | 320 | Sense-overlapping | SLC47A2 | ＜0.001 |
| Hsa_circ_0052318 | 7.24 | 23.65 | Down | chr19 | 2425 | Exonic | ZNF418 | ＜0.001 |
| Hsa_circ_0031482 | 6.54 | 23.32 | Down | chr14 | 3825 | Sense-overlapping | HECTD1 | ＜0.001 |
| circRNA_20084 | 6.10 | 21.40 | Down | chr2 | 313 | Sense-overlapping | METTL8 | ＜0.001 |
| circRNA_33356 | 6.12 | 20.80 | Down | chr8 | 2707 | Intergenic | - | ＜0.001 |
| Hsa_circ_0028123 | 5.04 | 17.96 | Down | chr12 | 318 | Sense-overlapping | ACACB | 0.002 |
| Hsa_circ_0004132 | 5.02 | 17.65 | Down | chr11 | 551 | Sense-overlapping | NAP1L4 | 0.002 |
| Hsa_circ_0025388 | 14.47 | 17.04 | Down | chr12 | 504 | Sense-overlapping | A2ML1 | ＜0.001 |
| circRNA_04921 | 5.04 | 17.01 | Down | chr10 | 472 | Sense-overlapping | CYP2C19 | 0.002 |
| circRNA_14622 | 4.79 | 16.84 | Down | chr17 | 381 | Sense-overlapping | EPN2 | 0.002 |
| circRNA_09750 | 38.93 | 16.81 | Down | chr13 | 636 | Sense-overlapping | SCEL | ＜0.001 |

**Supplementary Table 11** Association between hsa_circ_0063865 expression in esophageal cancer tissues and clinicopathological features

| Characteristics | Number | Hsa_circ_0063865 expression | | ***P*** value |
| --- | --- | --- | --- | --- |
|  |  | High | Low |  |
| Age |  |  |  |  |
| ≤60 | 21 | 10 | 11 | 0.954 |
| >60 | 70 | 36 | 34 |  |
| Gender |  |  |  |  |
| Male | 68 | 36 | 32 | 0.489 |
| Female | 26 | 11 | 15 |  |
| Smoking |  |  |  |  |
| Yes | 44 | 24 | 20 | 0.872 |
| No | 50 | 23 | 27 |  |
| Drinking |  |  |  |  |
| Yes | 37 | 19 | 17 | 0.935 |
| No | 57 | 28 | 29 |  |
| TNM |  |  |  |  |
| I/II | 39 | 11 | 28 | 0.005* |
| III/IV | 55 | 36 | 19 |  |
| Histological differentiation |  |  |  |  |
| Middle/High | 70 | 37 | 33 | 0.478 |
| Low | 24 | 10 | 14 |  |

**Supplementary Table 12. General characteristics of 110 ESCC patients**

| Parameters | Group | Number |
| --- | --- | --- |
| Age | <60 | 37 |
|  | ≥60 | 73 |
| Gender | Male | 75 |
|  | Female | 35 |
| Smoking | Yes | 66 |
|  | No | 44 |
| Drinking | Yes | 67 |
|  | No | 43 |

**Supplementary Table 13. General characteristics of 3 ESCC patients for the circRNA Sequencing**

| ID | Gender | Age | Clinical pathology |
| --- | --- | --- | --- |
| 1 | Female | 60 | poorly-differentiated |
| 2 | Female | 54 | poorly-differentiated |
| 3 | Female | 56 | poorly-differentiated |

**Supplementary Table 14. Primer sequences of circRNA and mRNAs**

| Gene | Type | Primer sequences | |
| --- | --- | --- | --- |
|  |  | Forward | Reverse |
| Hsa_circ_0063865 | Divergent | CCTCAGATGTAAAGTGTTATGACCAGC | AATGCATAGAAGGGCAAATCCATGATC |
|  | Convergent | CTGAGGCGGAGGAACCCTAAGG | AGCATCCCAGCACCAAATACAGAC |
| GAPDH |  | TGCACCACCAACTGCTTAGC | GGCATGGACTGTGGTCATGAG |
| RCN1 |  | CAACCAGAGCTTCCAGTACGA | TTCTGCACCCGTTTGATCCA |
| NMIIA |  | AGAGCTCACGTGCCTCAACG | TGACCACACAGAACAGGCCTG |
| eEF1A2 |  | GCCACCGTCAATAGGTGGAC | GATTCCGGAGCCGAGGTCTCA |

**Supplementary Table 15. SiRNA sequences of circRNA and mRNAs**

| Gene | No. | siRNA sequences | |
| --- | --- | --- | --- |
|  |  | Sense | Anti-sense |
| Hsa_circ_0063865 | 1 | GAAAGACAGCGGCUCAGGCA | UGCCUGAGCCGCUGUCUUUC |
|  | 2 | AGCGGCUCAGGCAGCUGAGG | CCUCAGCUGCCUGAGCCGCU |
|  | 3 | UCAGGCAGCUGAGGCGGAGG | CCUCCGCCUCAGCUGCCUGA |
| GAPDH | 1 | GUGGAGAUUGUUGCCAUCA | UGAUGGCAACAAUCUCCAC |
| RCN1 | 1 | CCGCAGAGUUUCAUGAUUC | GAAUCAUGAAACUCUGCGG |
| NMIIA | 1 | AGUCAGUGAUCAUUGCCUC | GAGGCAAUGAUCACUGACU |
| eEF1A2 | 1 | AAGAACAUGAUCACGGGUACA | UGUACCCGUGAUCAUGUUCUU |
| NC | 1 | UUCUCCGAACGUGUCACGU | ACGUGACACGUUCGGAGAA |

**Supplementary Table 16. Antibodies were used in this study**

| Antibodies | Source | Application | Dilution |
| --- | --- | --- | --- |
| NMIIA | Abcam | WB | 1:1000 |
| eEF1A2 | Abcam | WB | 1:1000 |
| RCN1 | Abcam | WB | 1:1000 |
| Nucleophosmin | Abcam | WB | 1:1000 |
| pPERK | Abcam | WB | 1:1000 |
| ATF4 | Abcam | WB | 1:1000 |
| peIF2α | Abcam | WB | 1:1000 |
| CHOP | Abcam | WB | 1:1000 |
| GAPDH | Abcam | WB | 1:1000 |
| HRP-linked Antibody | Abcam | WB | 1:5000 |
| pPERK | Abcam | IHC | 1:200 |
| ATF4 | Abcam | IHC | 1:200 |
| CHOP | Abcam | IHC | 1:200 |
| E-cadherin | Abcam | IHC | 1:200 |
| PCNA | Abcam | IHC | 1:200 |
